# *Trichoderma reesei* meiosis generates segmentally aneuploid progeny with higher xylanase-producing capability

**DOI:** 10.1186/s13068-015-0202-6

**Published:** 2015-02-25

**Authors:** Yu-Chien Chuang, Wan-Chen Li, Chia-Ling Chen, Paul Wei-Che Hsu, Shu-Yun Tung, Hsiao-Che Kuo, Monika Schmoll, Ting-Fang Wang

**Affiliations:** Taiwan International Graduate Program in Molecular and Cellular Biology, Academia Sinica, Taipei, 115 Taiwan; Institute of Life Sciences, National Defense Medical Center, Taipei, 115 Taiwan; Institute of Molecular Biology, Academia Sinica, Taipei, 115 Taiwan; Institute of Genome Sciences, National Yang-Ming University, Taipei, 112 Taiwan; Austrian Institute of Technology, Health and Environment Department, Bioresources, University and Research Center, UFT Campus Tulln, Tulln/Donau, 3430 Austria; Present address: Department of Forest Sciences, University of Helsinki, Helsinki, Finland

**Keywords:** *Trichoderma reesei*, *Hypocrea jecorina*, Genome evolution, Aneuploidy, Sexual development, Meiosis, Xylanase, Conidia pigmentation, Lignocellulosic biomass

## Abstract

**Background:**

*Hypocrea jecorina* is the sexual form of the industrial workhorse fungus *Trichoderma reesei* that secretes cellulases and hemicellulases to degrade lignocellulosic biomass into simple sugars, such as glucose and xylose. *H. jecorina* CBS999.97 is the only *T. reesei* wild isolate strain that is sexually competent in laboratory conditions. It undergoes a heterothallic reproductive cycle and generates CBS999.97(1-1) and CBS999.97(1-2) haploids with *MAT1-1* and *MAT1-2* mating-type loci, respectively. *T. reesei* QM6a and its derivatives (RUT-C30 and QM9414) all have a *MAT1-2* mating type locus, but they are female sterile. Sexual crossing of CBS999.97(1-1) with either CBS999.97(1-2) or QM6a produces fruiting bodies containing asci with 16 linearly arranged ascospores (the sexual spores specific to ascomycetes). This sexual crossing approach has created new opportunities for these biotechnologically important fungi.

**Results:**

Through genetic and genomic analyses, we show that the 16 ascospores are generated via meiosis followed by two rounds of postmeiotic mitosis. We also found that the haploid genomes of CBS999.97(1-2) and QM6a are similar to that of the ancestral *T. reesei* strain, whereas the CBS999.97(1-1) haploid genome contains a reciprocal arrangement between two scaffolds of the CBS999.97(1-2) genome. Due to sequence heterozygosity, most 16-spore asci (>90%) contain four or eight inviable ascospores and an equal number of segmentally aneuploid (SAN) ascospores. The viable SAN progeny produced higher levels of xylanases and white conidia due to segmental duplication and deletion, respectively. Moreover, they readily lost the duplicated segment approximately two weeks after germination. With better lignocellulosic biomass degradation capability, these SAN progeny gain adaptive advantages to the natural environment, especially in the early phase of colonization.

**Conclusions:**

Our results have not only further elucidated *T. reesei* evolution and sexual development, but also provided new perspectives for improving *T. reesei* industrial strains.

**Electronic supplementary material:**

The online version of this article (doi:10.1186/s13068-015-0202-6) contains supplementary material, which is available to authorized users.

## Background

Meiosis is a special type of cell division that gives rise to genetic diversity in sexually reproductive organisms. Programmed DNA double-strand breaks (DSBs) are spontaneously generated throughout the genome by the meiosis-specific Spo11 endonucleases in many organisms, such as yeast and mice [1]. In some fungi (for example, *Neurospora crassa* and *Coprinus cinereus*) meiotic DSBs are also induced via Spo11-independent mechanisms [2,3]. Both Spo11-dependent and Spo11-independent DSBs are repaired robustly by error-free homologous recombination to ensure genomic stability and accurate segregation of homologous chromosomes.

Previous studies also revealed that infertile or abnormal meiotic products are generated in some fungi [4]. For example, several fungi carry spore-killing meiotic drive elements [5], including *Neurospora sitophila*, *Neurospora intermedia*, *Podospora anserina*, and *Cochliobolus heterostrophus*. Recently, two Spore killer elements (*Sk-2* and *Sk-3*) were reported to be located near a chromosome rearrangement site in *Neurospora crassa* [6]. It is still unclear whether chromosome rearrangement can cause meiotic drive. The molecular mechanism of the Spore killer in *Neurospora crassa* is still a mystery. Plant pathogenic fungi, for example, *Mycosphaerella graminicola* (anamorph *Septoria tritici*) and *Nectria haematococca* mating population VI (anamorph *Fusarium solani*), generate ascospores containing “extra” chromosomes, termed “conditionally dispensable” chromosomes [7-11]. In the human fungal pathogen *Cryptococcus neoformans*, a large segmental duplication occurs during meiosis via telomere-telomere fusion and chromosomal translocation between two different chromosomes [12,13]. The hybrid infertility of two closely related *Schizosaccharomyces* strains (*S. pombe* and *S. kambucha*) resulted from the chromosome rearrangement of their genomes [14]. Therefore, genome heterozygosity may be a cause of the production of segmental aneuploidy (SAN) and whole chromosome aneuploidy (WCA) ascospores in fungal meiosis.

SAN and WCA are often deleterious for survival due to the altered gene dosage [15]. However, they sometimes provide benefits in fitness to cells under stress; for instance, the *SUL1* gene is amplified in budding yeast cells cultured in sulfate-depleted medium for generations [16]. Budding yeast cells also exhibit SAN or WCA after being cultured at high temperature for over 490 generations [17] or when continuously cultured in copper-containing medium [18]. Notably, these strains readily exhibit return to euploidy (RTU) after the relief of stress-inducing conditions, indicating that genome plasticity is an adaptive strategy to gain transient advantages [15-18].

*Trichoderma* is a genus of ascomycete fungi that is present in soils and in other diverse habitats [19]. These fungi are beneficial symbiotic partners for plants, particularly crops. *Trichoderma* spp. secrete cellulases and hemicellulases that degrade β-glucan and xylan, the key structural components of lignocellulosic biomass, to produce glucose and xylose, respectively. Several cellulase-overproducing mutants (such as RUT-C30 and QM9414) derived from the *Trichoderma reesei* QM6a isolate have been widely used in industrial applications [20-23]. Due to multiple rounds of chemical and/or physical mutagenesis, the genomes of these hypersecretion mutants have numerous mutations, deletions, and DNA rearrangements [20,24,25]. Currently, the major bottleneck of lignocellulosic biomass degradation is enzyme cost, and these industrial strains secrete less xylan-degrading hemicellulases than β-glucan-degrading cellulases. *T. reesei* is the anamorph of the pantropical ascomycete *Hypocrea jecorina* [26]*.* The *H. jecorina* CBS999.97 wild isolate undergoes a heterothallic reproductive cycle, generating CBS999.97(1-1) and CBS999.97(1-2) haploids with *MAT1-1* and *MAT1-2* mating-type loci, respectively. QM6a has a *MAT1-2* mating type locus, but it is female sterile. Sexual crossing of the CBS999.97(1-1) haploid with the CBS999.97(1-2) haploid or QM6a(1-2) yields fruiting bodies that contain asci with 16-part ascospores [27]. This sexual crossing approach has opened up new perspectives for these biotechnologically important fungi.

Here, we report that there is a reciprocal exchange (*re*) between two scaffolds in CBS999.97(1-1) compared with other *Trichoderma* wild isolates. Herein, the two CBS999.97 haploid strains and QM6a are referred as to as CBS999.97(1-1, *re*), CBS999.97(1-2, *wt* [*wild type*]), and QM6a(1-2, *wt*), respectively. Due to genome heterozygosity, sexual crossing of CBS999.97(1-1, *re*), CBS999.97(1-2, *wt*), or QM6a(1-2, *wt*) frequently generated equal numbers of inviable and viable SAN ascospores.

## Results

### The 16 ascospores in *T. reesei* asci are generated via meiosis followed by two rounds of postmeiotic mitosis

**Figure 1:**
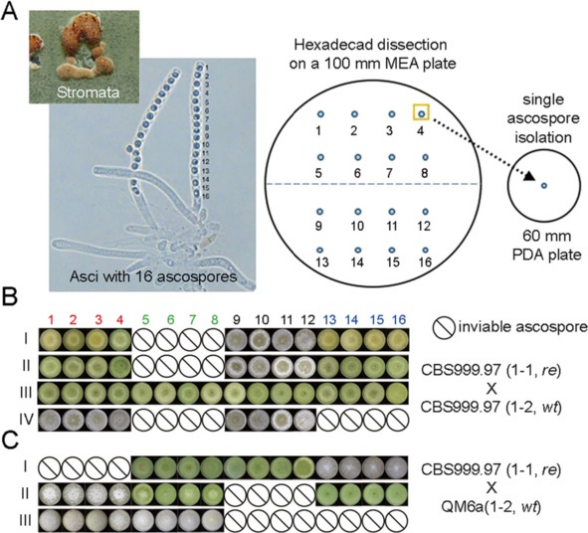
**Hexadecad dissection. (A)** Upper panel, stromata; lower panel, developing asci of which two contain 16 ascospores. Each ascospore is numbered according to its order in the ascus. Sixteen ascospores from a hexadecad were sequentially separated and grown on individual 100-mm malt extract agar (MEA) plates. A single colony from one ascospore was isolated and transferred individually to a 60-mm potato dextrose agar (PDA) plate to determine the spore viability, spore color, and colony morphology. **(B-C)** Ascospore phenotype of hexadecads from the sexual crossing of the wild isolate CBS999.97(1-1, *re*) with CBS999.97(1-2, *wt*) (n ≥ 20) or QM6a(1-2, *wt*) (n ≥ 10). Hexadecads were grown in constant darkness. Sixteen single-ascospore colonies were aligned sequentially according to the ascospore order. The inviable ascospores are indicated by a black circle with a cross.

CBS999.97 is the only *T. reesei* wild isolate strain that is sexually competent in laboratory conditions [27]. Sexual crossing of CBS999.97(1-1, *re*) with CBS999.97(1-2, *wt*) or QM6a (1-2, *wt*) yields fruiting bodies containing asci with 16 ascospores (that is, hexadecads). To reveal the molecular mechanism of *T. reesei* sexual development, we applied the yeast tetrad dissection technique to sequentially separate the 16 ascospores from an ascus (Figure 1A). Each ascospore was individually cultured, and the spore viability, colony morphology, and colony color were determined (Figure 1B and 1C). Genomic PCR genotyping (Additional file 1: Figure S1 and Additional file 2: Table S1) of all 16 ascospores from one hexadecad (Figure 1B, III) further revealed that each hexadecad was classified into four linearly arranged groups, and each group contained four genetically indistinguishable ascospores. Finally, by staining the developing asci with 4',6-diamidino-2-phenylindole (DAPI), we also visualized two meiotic divisions and two further mitotic divisions (Additional file 3: Figure S2). Together, these results indicate that the 16 ascospores are generated via meiosis followed by two rounds of postmeiotic mitosis.

### The CBS999.97 wild isolate frequently generates abnormal sexual progeny

QM6a(1-2, *wt*), CBS999.97(1-2, *wt*), and CBS999.97(1-1, *re*) all propagate mycelia and produce green conidia (that is, asexual spores). We found that sexual crossing of CBS999.97(1-1, *re*) with CBS999.97(1-2, *wt*) (Figure 1B) or QM6a(1-2, *wt*) (Figure 1C) often yielded hexadecads (>90%) with abnormal ascospores. The majority of the hexadecads (>80%, n ≥ 30) contained only 12 viable ascospores: eight of them germinated and then produced green conidia, whereas the other four produced white conidia (Figure 1B, I and II). The inviable ascospores were unable to germinate. Only approximately 10% of hexadecads could generate 16 viable ascospores, and these ascospores germinated and produced green conidia (Figure 1B, III). Finally, the remaining approximately 9% of the hexadecads produced eight inviable ascospores as well as an equal number of viable, white-conidia ascospores (Figure 1B, IV).

**Figure 2:**
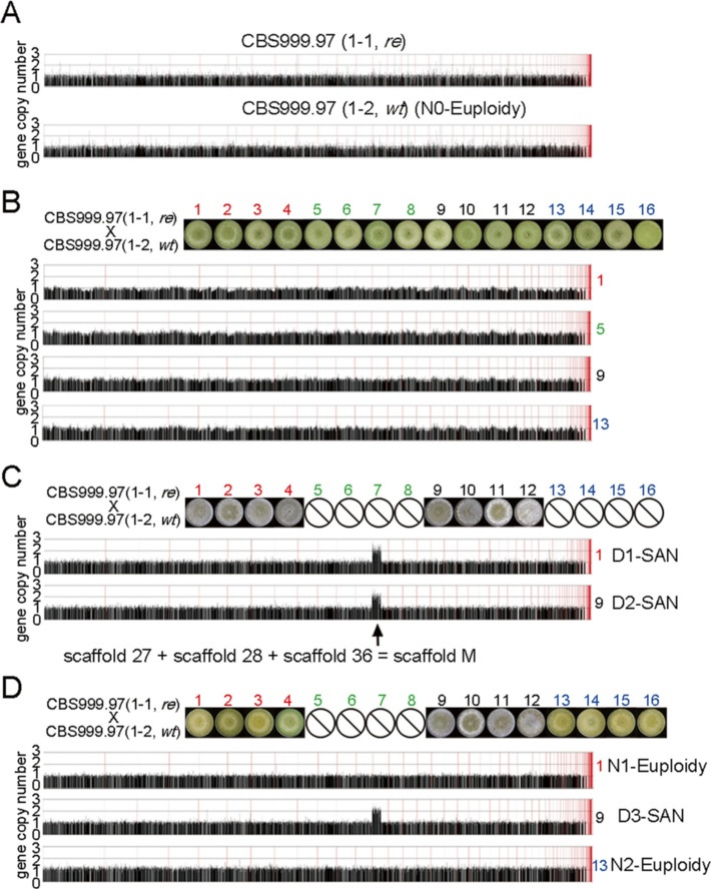
**aCGH. (A)** Gene copy number of the two parental wild isolate haploids. CBS999.97(1-2, *wt*) genomic DNA was used as a reference to measure DNA copy number changes of CBS999.97(1-1, *re*) and *vice versa*. Each line in the histogram represents one oligonucleotide and its position in the CBS999.97(1-2, *re*) and CBS999.97(1-2, *wt*) genomic sequence. The CBS999.97(1-2, *wt*) genome, such as that of QM6a(1-2, *wt*) [25], comprises 89 scaffolds. Moreover, three contiguous scaffolds (27, 28, 36) were reassembled into a much larger scaffold, scaffold M. The length of scaffold M is slightly shorter than that of scaffold 11. Normalized means of gene copy number for the oligonucleotides covering the 87 scaffolds are shown. The 87 scaffolds are ordered from left to right according to their length. **(B)** Gene copy number of four representative ascospores (numbers 1, 5, 9, and 13) of asci III (Figure 1B) with 16 viable ascospores. **(C)** Gene copy number of two representative ascospores (numbers 1 and 9) of asci IV (Figure 1B) with eight inviable ascospores. **(D)** Gene copy number of two representative ascospores (numbers 1, 9, and 13) of asci I (Figure 1B) with 12 inviable ascospores. The three strains with duplicated segments (D1-SAN, D2-SAN, and D3-SAN) and the three euploid control strains (N0-Euploidy, N1-Euploidy, and N2-Euploidy) are also indicated.

**Figure 3:**
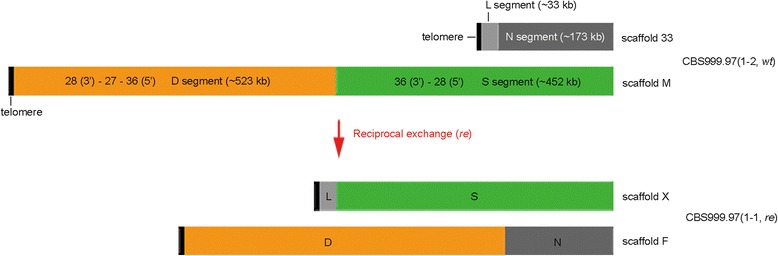
**Reciprocal exchange between scaffold M and scaffold 33.** Scaffold M and scaffold 33 in CBS999.97(1-2, *wt*), and scaffold F and scaffold X in CBS999.97(1-1, *re*). Organization and length of the four segments (L, N, D, and S) in these four scaffolds are indicated.

The *T. reesei* v2.0 database (Department of Energy, Joint Genome Institute, USA) of the QM6a(1-2, *wt)* genome comprises 89 scaffolds and 97 contigs [25]. Using several commercially available cell-wall digesting enzymes (such as β-glucanase, Driselase, and lyticase), we were unable to prepare intact CBS999.97 and QM6a chromosomes for clamped homogeneous electric field (CHEF) gel electrophoresis. Novozyme 234 was previously used to prepare *Trichoderma* protoplasts [28] and intact chromosomes for CHEF analysis [29,30], but it has not been commercially available since 2000 [31]. Instead, we applied the microarray-based comparative genomic hybridization (aCGH) technique to identify genome-wide gene copy-number variation. Comparing the aCGH data between the two CBS999.97 parental haploid strains, their genome-wide gene copy numbers seemed to be equivalent, suggesting that both strains are euploid (Figure 2A). We then used the CBS999.97(1-2, *wt*) as the reference genome in further analyses. The ascospores with green-conidia phenotype were all euploid (Figure 2B and D). Notably, all white-conidia progeny generated from either the eight viable ascospore hexadecad (Figure 2C) or the 12 viable ascospore hexadecad (Figure 2D) were SAN with a copy gain within scaffold 27 (130 genes and about 431 kb), the 3′ terminus of scaffold 28 (12 genes, about 40 kb), and the 5′ terminus of scaffold 36 (15 genes, about 52 kb) (Additional file 4: Figure S3A). This approximately 523-kb duplicated region was referred to as the “duplicated (D) segment” (Figure 3).

Deep sequencing of the CBS999.97(1-2, *wt*) genome (http://bc.imb.sinica.edu.tw/~lab229/Text_file_T1-4.rar) and genomic PCR analyses (Additional file 4: Figures S3B and S3C) revealed that three scaffolds (27, 28, and 36) were contiguous segments and were together referred to as scaffold “M” (Figure 2C and Figure 3). Accordingly, we further referred to the about 452-kb unduplicated region of scaffold M as the “single (S) segment” (Figure 3). Genomic PCR and sequencing results also revealed that a repeated hexanucleotide sequence TTAGGG, which is the telomeric repeat of QM6a(1-2, *wt*) [25], is connected to the 3′ terminus of scaffold 28. This finding provides evidence that the telomere is located near the 5′ terminus of scaffold M (Figure 3).

### Identification of the chromosome rearrangement region in CBS999.97 wild isolate strains

The ascospore phenotype of our study seemed to be similar to that of the hybrid infertility of *S. pombe* and *S. kambucha* [14]. Zanders *et al.* indicated that chromosome rearrangement leads to genome heterozygosity of the two closely related haploid strains, and the meiotic recombination between the two homoeologous chromosomes generates both inviable and viable SAN progeny [14]. Our aCGH results also supported this conclusion. The duplication of the D segment in the viable SAN ascospores apparently resulted from a chromosome rearrangement region located in the second intron of a novel gene (ID 112288; scaffold 36:54323-54324 bp) (Additional file 5: Figure S4A). Additional PCR and sequencing analysis showed that, compared to QM6a(1-2, *wt*) [25], the first intron of 112288 was deleted in both CBS999.97(1-2, *wt*) (Additional file 5: Figure S4A) and CBS999.97(1-1, *re*) (Additional file 5: Figure S4B). Moreover, compared with the genomes of CBS999.97(1-2, *wt)* and QM6a(1-2, *wt*), a large chromosome translocation was found at this region of the CBS999.97(1-1, *re*) genome: (1) the 5′ terminus of scaffold 33 (1-33,249 bp; referred to as the “L” segment) links the S segment of scaffold M to form a new scaffold “X” in CBS999.97(1-1, *re*); and (2) the 3′ terminus of scaffold 33 (33,250-207,997 bp, referred to as the “N” segment) and the D segment of scaffold M physically link and form a new scaffold “F” in CBS999.97(1-1, *re*) (Figure 3, (http://bc.imb.sinica.edu.tw/~lab229/Text_file_T1-4.rar) and Additional file 5: Figure S4B).

### Genome heterozygosity is responsible for the production of SAN ascospores

To confirm whether this reciprocal exchange (*re*) allele is the cause of meiotic drive segmental duplication, we identified two new haploids, CBS999.97(1-1, *wt*) and CBS999.97(1-2, *re*), from the offspring of the two parental haploids, CBS999.97(1-1, *re*) and CBS999.97(1-2, *wt*) (Additional file 1: Figure S1, Additional file 5: Figure S4C and S4D). We found that all 16 ascospores generated from sexually crossing CBS999.97(1-1, *re*) with CBS999.97(1-2, *re*) or CBS999.97(1-1, *wt*) with CBS999.97(1-2, *wt*) were viable. In addition, they all germinated to form mycelia with green conidia (Table 1). These results indicate that the genome heterozygosity is the primary cause for production of the inviable ascospores as well as the viable, white-conidia ascospores.

**Table 1:** **The chromosome reciprocal exchange (** ***re*** **) allele, but not NHEJ genes (** ***tku70*** **and** ***tmus53*** **), is responsible for the formation of meiotic drive SAN progeny**

**Strain background**	**Sexual**	**Number of asci with 4 or 8 inviable ascospores**	**Number of asci dissected**
	**crossing**		
CBS999.97	(1-1, *re*) x (1-2, *wt*)	19	20
	(1-1, *wt*) x (1-2, *wt*)	0	10
	(1-1, *re*) x (1-2, *re*)	0	10
CBS999.97 *tku70*Δ	(1-1, *re*) x (1-2, *wt*)	8	10
	(1-1, *wt*) x (1-2, *wt*)	0	10
	(1-1, *re*) x (1-2, *re*)	0	10
CBS999.97 *tmus53*Δ	(1-1, *re*) x (1-2, *wt*)	10	10
	(1-1, *wt*) x (1-2, *wt*)	0	10
	(1-1, *re*) x (1-2, *re*)	0	10

Next, we examined whether the non-homologous end joining (NHEJ) DNA repair pathway is responsible for the production of inviable ascospores. Two NHEJ proteins, Ku70 and Ku80, form a heterodimer and function as a molecular scaffold at DSB ends to which other NHEJ proteins (such as the DNA ligase IV, Lig4) can bind [32]. The *T. reesei* genes that encode Ku70, Ku80, and Lig4 were previously referred to as *tku70*, *tku80*, and *tmus53*, respectively [33,34]. We found that deletion of either *tku70* or *tmus53* did not affect meiosis, ascospore number, or ascospore viability in any of the relevant strains (Table 1 and Additional file 5: Figure S4). These results suggest that homologous recombination (or crossover) during meiotic prophase or random chromosome segregation during the meiotic nuclear division (MI) may be responsible for the production of SAN ascospores.

### Interhomolog recombination between two homoeologous chromosomes accounts for most, if not all, SAN ascospores during meiosis

**Figure 4:**
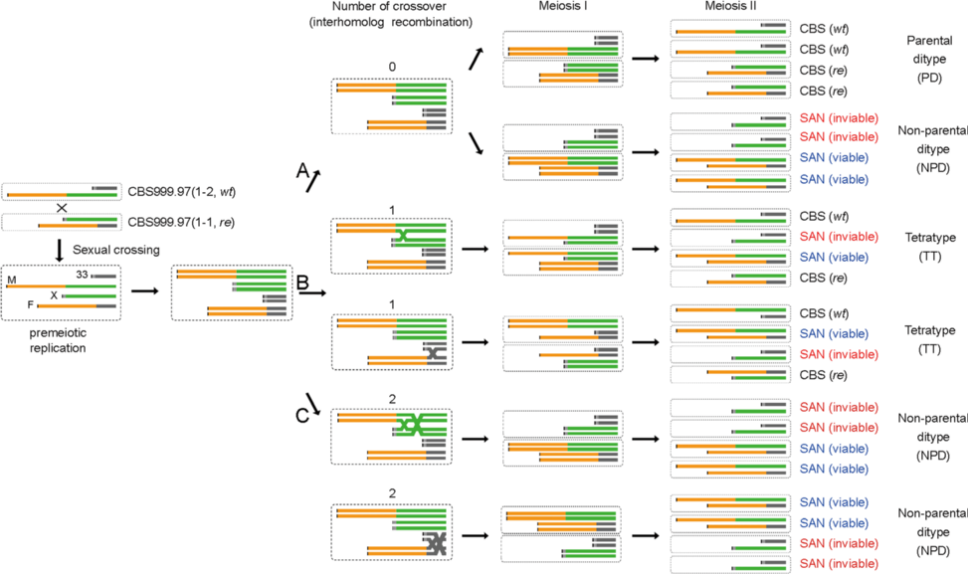
**Meiotic crossover and chromosome segregation are responsible for the formation of segmental aneuploidy.** The hypothetical model of meiotic drive viable and inviable ascospores. **(A)** In the absence of crossover (interhomolog recombination), the parental ditype (PD) hexadecad asci and the non-parental ditype (NPD) hexadecad asci can be generated by random segregation of homologous chromosomes during the first meiotic nuclear division (MI). **(B)** A single crossover between scaffold M and scaffold F or scaffold X and scaffold 33 results in the tetratype (TT) hexadecad asci. **(C)** The NPD hexadecad asci can also be generated if two crossovers exist between scaffold M and F or scaffold X and 33.

**Figure 5:**
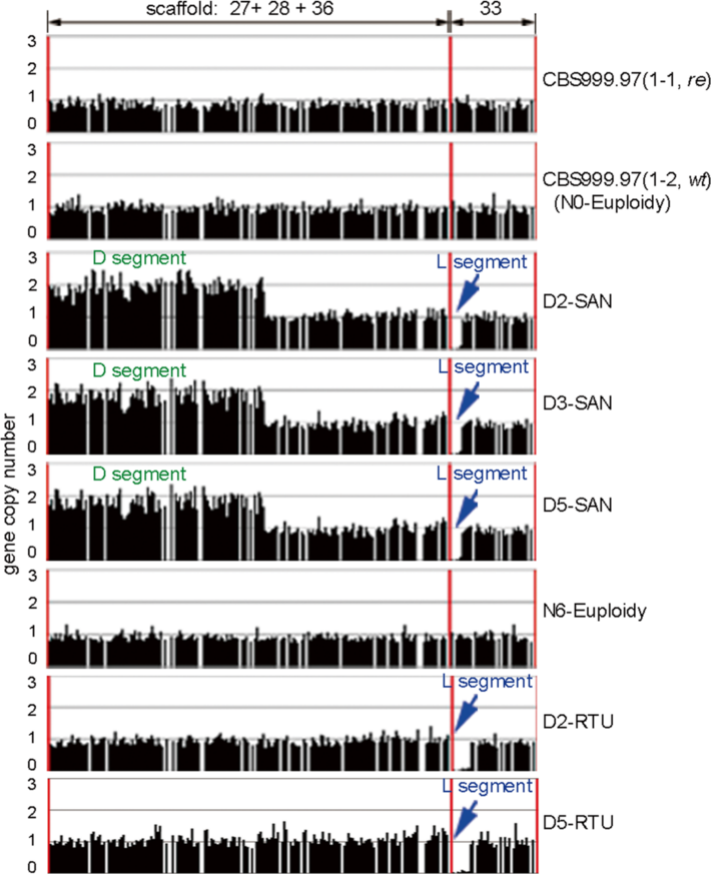
**The SAN mutants contain a duplicated D segment but no L segment.** Shown are aCGH results in scaffold M (27 + 28 + 36) and scaffold 33 of the two parental strains (upper two panels), three viable SAN progeny (D2, D3, and D5), and one viable euploid progeny (N6) (middle four panels), and the return-to-euploid (RTU) strains of D2-SAN and D5-SAN (D2-RTU and D5-RTU, lower two panels). The three SAN progeny contain two D segments and lose the L segment, whereas the D2-RTU and D5-RTU strains have a D segment but no L segment.

We propose a hypothetical model (Figure 4) to explain the sexual crossing results in Figure 1. First, the hexadecads with 16 viable ascospores were referred to as “parental ditype (PD)”; all of these euploid ascospores could germinate and produce green conidia. Eight of them, such as CBS999.97(*wt*), have scaffold M and scaffold 33; the other eight ascospores, such as CBS999.97(*re*), have scaffold F and scaffold X. Second, the hexadecads with eight white-conidia SAN ascospores and eight inviable SAN ascospores were referred to as “non-parental ditype (NPD)”; these viable, white-conidia SAN ascospores contain two D segments but no L segment (Figures 2C and 5). Accordingly, we inferred that the inviable SAN ascospores have two L segments but no D segment. Finally, the hexadecads with four inviable ascsospores and 12 viable ascospores were referred to as “tetratype (TT)”, containing four euploid ascospores with scaffold M and scaffold 33, four euploid ascospores with scaffold F and scaffold X, four viable SAN ascospores with two D segments but no L segment, and four inviable SAN ascospores with two L segments but no D segment (Figures 2D and 5).

When no interhomolog recombination (or crossover) occurs between the four scaffolds (M, 33, X, and F) during meiotic prophase, PD and NPD can be produced simply by random chromosome segregation during MI (Figure 4A). In contrast, TT is likely generated via a single crossover between scaffold M and scaffold X or between scaffold 33 and scaffold F (Figure 4B). NPD may also be generated when two crossovers occur between two of these four scaffolds (Figure 4C). Because our single ascospore isolation experiments (Figure 1) revealed that there were more TT (>80%) than PD (about 10%) and NPD (about 9%), we suggest that meiotic recombination occurs at a high frequency between scaffold M and scaffold X or between scaffold 33 and scaffold F.

Notably, the hypothesis described here is consistent with our aCGH and genetic results: first, all three viable SAN ascospores we examined (D2, D3, and D5) had two D segments but no L segment (Figure 5); second, we inferred that the inviable SAN ascospores have two L segments but no D segment. The D segment is about 523 kb in length and contains at least 113 genes (Additional file 6: Table S2), including several putative essential genes such as an actin-like protein (ID 111468). Due to the lack of D segments, these inviable SAN ascospores failed to germinate.

### The ancestral *T. reesei* genome is similar to that of CBS999.97(1-2, *wt*)

The sexually competent CBS999.97 strain was isolated from a storage lake in French Guiana [35], whereas QM6a(1-2, *wt*) was collected on the Solomon Islands during the Second World War [36]. Several other non-CBS999.97 haploid strains were isolated from different geographical locations [27]. We then applied the same diagnostic PCR method (Additional file 5: Figure S4C) to study the distribution of the four scaffolds (M, 33, F, X) in nine representative non-CBS999.97 isolates collected from French Guiana, Brazil, Indonesia, and New Caledonia. Using the four primers (A-D) described above (Additional file 2: Table S1 and Additional file 5: Figure S4C), we were able to detect scaffold 33 in eight isolates (except G.J.S. 84-473) and scaffold M in six isolates (except G.J.S. 84-473, G.J.S. 86-410, and G.J.S. 93-23) (Additional file 7: Table S3). Intriguingly, scaffold F and scaffold X were not detected in any of the nine non-CBS999.97 isolates, indicating that the genomes of these non-CBS999.97 isolates might be more similar to those of CBS999.97(1-2, *wt*) or QM6a(1-2, *wt*) than that of CBS999.97(1-1, *re*).

This hypothetical model was further confirmed by sexual crossing and single ascospore isolation experiments between those wild isolates and CBS999.97 strains (Table 2). CBS999.97(1-2, *wt*) sexually crossed with the three French Guiana *MAT1-1* isolates, G.J.S. 86-404(1-1, *wt*), G.J.S. 86-410(1-1, *wt*), and G.J.S. 84-473(1-1, *wt*), generated asci with 16 viable ascospores, although our diagnostic PCR method failed to detect scaffold M and scaffold 33 in G.J.S. 84-473(1-1, *wt*). Asci with 16 viable ascospores were also generated when CBS999.97(1-2, *wt*) was crossed with G.J.S. 85-249(1-1, *wt*) (Indonesia, Celebes) or when CBS999.97(1-1, *wt*) was crossed with four *MAT1-1* wild isolates, including G.J.S. 89-7(1-1, *wt*) (Brazil, Para), G.J.S. 97-178(1-2, *wt*) (Brazil, Para), G.J.S. 93-23(1-2, *wt*) (New Caledonia), and G.J.S. 85-236(1-2, *wt*) (Indonesia, Celebes). In contrast, asci with four or eight inviable ascospores were frequently generated when CBS999.97(1-2, *re*) was sexually crossed with three different wild isolates, G.J.S. 86-404(1-1, *wt*; French Guiana), G.J.S. 86-410(1-1, *wt*; French Guiana), and G.J.S. 85-249(1-1, *wt*; Indonesia, Celebes). Similarly, asci with four or eight inviable ascospores were also generated when CBS999.97(1-1, *re*) was sexually crossed with four different wild isolates, G.J.S. 89-7(1-2, *wt*; Brazil, Para), G.J.S. 97-178(1-2, *wt*; Brazil, Para), G.J.S. 93-23(1-2, *wt*; New Caledonia), and G.J.S. 85-236(1-2, *wt*; Indonesia, Celebes). Therefore, we inferred that the ancestral *T. reesei* genomes likely contain scaffold M and scaffold 33, and scaffold F and scaffold X evolved later in French Guiana via an unequal DNA rearrangement between scaffold M and scaffold 33. Intriguingly, these non-CBS999.97 isolates examined here could only sexually cross with the haploid progeny generated from the CBS999.97 wild isolate strain but not with each other (Additional file 8: Table S4).

**Table 2:** **Sexual crossing of CBS999.97 with non-CBS999.97 isolates**

**Sexual crossing**	**Number of asci dissected**	**Number of asci with 4 or 8 inviable ascospores**
CBS999.97(1-2, *wt*; French Guiana) & G.J.S. 86-404(1-1, *wt*; French Guiana)	7	0
CBS999.97(1-2, *wt*; French Guiana) & G.J.S. 86-410(1-1, *wt*; French Guiana)	8	0
CBS999.97(1-2, *wt*; French Guiana) & G.J.S. 84-473(1-1, *#*; French Guiana)	7	0
CBS999.97(1-1, *wt*; French Guiana) & G.J.S. 89-7(1-2, *wt*; Brazil, Para)	7	0
CBS999.97(1-1, *wt*; French Guiana) & G.J.S. 97-178(1-2, *wt*; Brazil, Para)	9	0
CBS999.97(1-1, *wt*; French Guiana) & G.J.S. 93-23(1-2, *#*; New Caledonia)	9	0
CBS999.97(1-1, *wt*; French Guiana) & G.J.S. 85-236(1-2, *wt*; Indonesia Celebes)	9	0
CBS999.97(1-1, *wt*; French Guiana) & G.J.S. 85-249(1-1, *wt*; Indonesia Celebes)	9	7
CBS999.97(1-2, *re*; French Guiana) & G.J.S. 86-404(1-1, *wt*; French Guiana)	9	8
CBS999.97(1-2, *re*; French Guiana) & G.J.S. 86-410(1-1, *wt*; French Guiana)	7	5
CBS999.97(1-1, *re*; French Guiana) & G.J.S. 89-7(1-2, *wt*; Brazil, Para)	9	8
CBS999.97(1-1, *re*; French Guiana) & G.J.S. 97-178(1-2 *wt*; Brazil, Para)	8	6
CBS999.97(1-1, *re*; French Guiana) & G.J.S. 93-23(1-2, *#*; New Caledonia)	8	4
CBS999.97(1-1, *re*; French Guiana) & G.J.S. 85-236(1-2, *wt*; New Caledonia)	7	6

#: Diagnostic PCR failed to detect M, F, 33 or X in G.J.S. 84-473 and G.J.S. 93-23

### The viable SAN progeny exhibit return to euploidy (RTU) in vegetative growth

**Figure 6:**
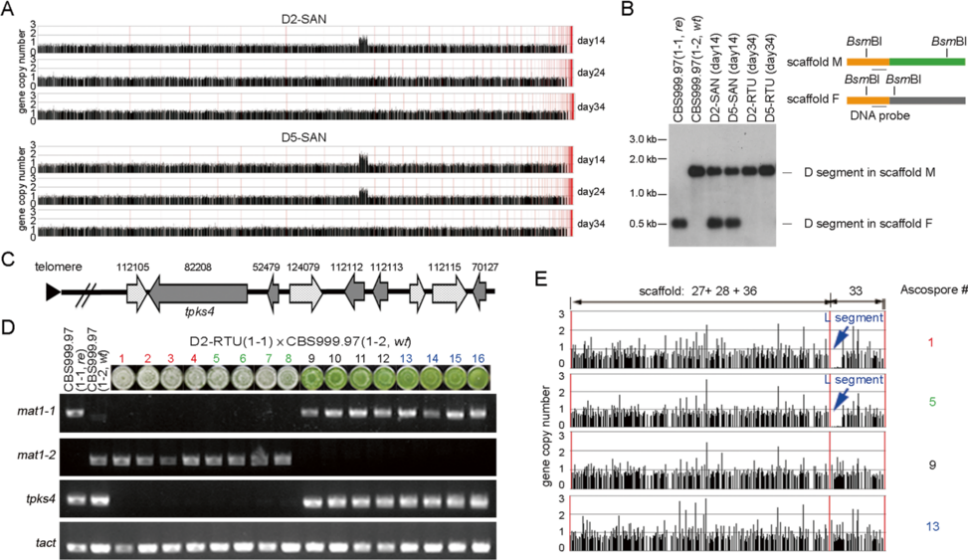
**Loss of the L segment leads to a white colony of ascospores. (A)** The genome stability of two different SAN strains (D2-SAN and D5-SAN) during vegetative growth was examined. The number of days post ascospore germination in a dextrose-containing malt extract agar (MEA) medium is indicated on the right. The Gene Expression Omnibus accession number is GSE-42359. **(B)** Southern blot analysis of the two D segments before and after RTU. The locations of the *BsmB*I restriction enzyme sites and the DNA probe used for Southern blot analysis are indicated in the right panel. The restriction fragments of the D segments in scaffold F and scaffold M are about 400 bp and 1,500 bp in length, respectively. The two parental haploid strains, CBS999.97(1-2, *re*) and CBS999.97(1-2, *wt*), were used as positive controls. **(C)** The organization and protein ID numbers of the nine annotated genes in the L segment. *T. reesei* polyketide synthase (*tpks4*) gene has been shown to be responsible for the formation of green conidia [37]. **(D)** Loss of the L segment or *tpks4* led to the white-conidia phenotype. Twenty hexadecads generated from sexual crossing of CBS999.97(1-2, *wt*) with D2-RTU or D5-RTU were dissected and isolated as described in Figure 1A. These 20 hexadecads all generated 16 ascospores. After germination, eight produced white conidia and the other eight produced green conidia. Shown are the conidia-color phenotype and genotyping data (by genomic PCR) of a representative hexadecad with 16 viable ascospores. (**E**) The aCGH results of four representative ascospores (numbers 1, 5, 9, and 16) in (D). The two white-conidia progeny (numbers 1 and 5) have no L segment, whereas the two green-conidia progeny (numbers 9 and 16) have the L segment.

Several studies have suggested that the duplicated regions are not stable and are usually lost after several generations [15-18]. To test the stability of our SANs, they were continuously cultured in rich MEA medium and the genome-wide gene copy number was analyzed every ten days. The aCGH data showed that only one D segment was detected after 34 days of vegetative growth (Figure 6A). The results of physical analysis (n = 10) also confirmed that the D segment from scaffold F was lost during vegetative growth (Figure 6B), suggesting that the D segment in scaffold F is highly unstable compared to the one in scaffold M. Therefore, these RTU strains have genomes similar to CBS999.97 (1-2, *wt*) but lack the L segments (Figure 5).

### Loss of the L segment resulted in a white-conidia phenotype

The viable SAN progeny exhibited a white-conidia phenotype before (Figure 1) and after RTU (Figure 6A). The *T. reesei* polyketide synthase 4 gene (*tpks4*) has been reported to be responsible for the green conidial pigmentation, and the *tpks4*Δ mutant produces white conidia [37]. Notably, *tpks4* is one of the nine annotated genes in the L segment (Figure 6C). We inferred that deletion of the L segment, but not D segment duplication, may lead to the white-conidia phenotype. To further determine whether the loss of the L segment is the only factor responsible for the white-conidia phenotype, we backcrossed the RTU strains with the parental strain CBS999.97(1-2, *wt*). The 16 offspring of the backcross showed an eight green-conidia and eight white-conidia phenotype (Figure 6D), supporting our hypothesis. Genomic PCR genotyping experiments further showed that only the green-conidia progeny but not the white-conidia progeny have the *tpks4* gene (Figure 6D). Finally, the aCGH results of the representing progeny confirmed that the green-conidia ascospore but not the white-conidia ascospore had the L segment (Figure 6E). These data imply that lack of *tpks4* is responsible for the white-conidia phenotype.

### The viable SAN progeny showed high hemicellulase-producing capability

**Figure 7:**
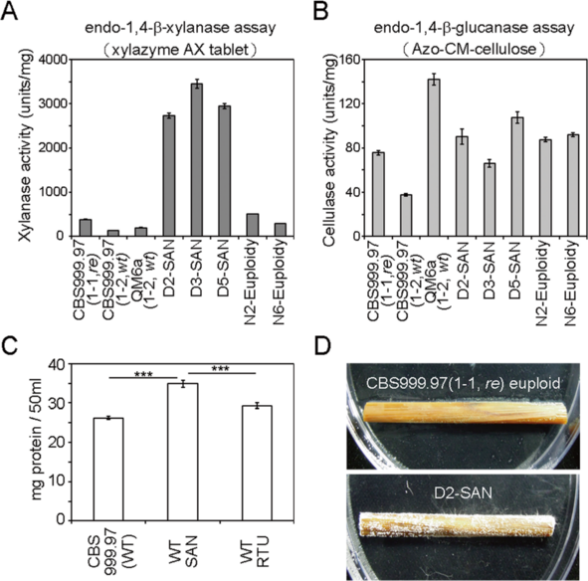
**The SAN progeny produce higher levels of xylanases. (A)** Xylanase and **(B)** cellulase specific activities (U/mg of mycelium) of the indicated strains were measured using xylazyme AX tablet and Azo-CM-cellulose as substrates, respectively. Experiments were conducted in triplicate and are presented with standard deviations. **(C)** The SAN strains produced more biomass than the two CBS999.97 euploid strains or the RTU strains in a xylan-based Mandels-Andreotti medium. Experiments were conducted with two different colonies, each in triplicate, and are presented with mean values ± SEM (error bars). **(D)** The D2-SAN grew better than the parental euploid strain CBS999.97(1-1, *re*) on rice straw.

A hallmark of the QM6a(1-2, *wt*) genome is that many genes encoding the carbohydrate-active enzymes (CAZymes) are non-randomly distributed in several gene clusters [25]. The CAZymes can cleave, build, and rearrange oligo- and polysaccharides [38]. The majority of the CAZyme genes in these clusters encode glycoside hydrolases that contribute to the degradation of lignocelluloses and plant cell walls. Previous transcriptomic studies also indicated that adjacent or nearly adjacent genes were coexpressed in four CAZyme gene clusters located in scaffolds 1, 6, 28, and 29 [25]. Notably, the CAZyme gene cluster in scaffold 28 is located at the D segment (Additional file 6: Table S2), including an endo-β-1,4-xylanase gene (ID 69276), a β-mannosidase (ID 69245), and the *cip2* glucuronoyl esterase gene (ID 123940). The D segment also contains the *xyn2* xylanase II gene in scaffold 27 (ID 123818). These four genes all encode enzymes with hemicellulase activity [38]. Indeed, the SAN progeny with two D segments exhibited a higher xylanase activity than the euploid progeny or their parental haploid strains (Figure 7A).

*T. reesei* is well known as an industrial workhorse for synthesizing cellulases and hemicellulases [25], and we therefore wanted to know if the SANs also showed enhancement in cellulase activity. The cellulase activity assay showed that compared with the wild-type control, the SANs did not produce higher levels of cellulases (Figure 7B). These data suggest that the production of cellulase and xylanase is differentially regulated in these SAN progeny.

### The viable SAN progeny showed growth advantage

Although variations in chromosome copy number are often detrimental to organisms [15], studies in several microorganisms also indicate that DNA copy-number alterations can be beneficial, increasing survival under selective pressure [16-18]. When grown on a xylan-based medium, the SAN strains produced more biomass than either the parental CBS999.97 euploid strains or the RTU strains (Figure 7C). Given the lack of L segment in the RTU strains, we conclude that D segment duplication, but not L segment deletion, is responsible for the growth advantage in xylan-based media. Intriguingly, the SAN strain also grew better than the CBS999.97 euploid haploid on rice straw (Figure 7D). Taken together, these results suggest that meiotic drive segmental duplication apparently provides an advantage to transiently enhance the efficiency for degrading and utilizing lignocellulosic biomass.

### Applying the sexual crossing strategy to improve industrial strains

To further improve the xylanase production activity of two cellulase-overproducing mutants, RUT-C30(1-2, *wt*) and QM9414(1-2, *wt*), we tried to generate SANs under both backgrounds. However, sexual crossing of CBS999.97(1-1, *re*) with either RUT-C30(1-2, *wt*) or QM9414(1-2, *wt*) usually produced asci or hexadecads with no or only four viable ascospores (Additional file 9: Figure S5). Because the genomes of these two cellulase-overproducing mutants acquired numerous mutations, deletions, and chromosomal rearrangements via multiple rounds of physical and chemical mutagenesis [20,24], genome heterozygosity could also account for the meiotic drive lethality we observed here. Our results here suggest that sexual crossing should be more cautiously used to improve these industrially used hypersecretion mutants.

## Discussion

The current study reveals several novel characteristics of *T. reesei* sexual development and genome plasticity. First, *T. reesei* generates asci with 16-part ascospores via meiosis and two further rounds of mitotic nuclear divisions. Second, our data suggest that the genome of ancestral *T. reesei*, such as that of CBS999.97(1-2, *wt*) and QM6a(1-2, *wt*), contains scaffold M and scaffold 33. Scaffold X and scaffold F were generated via a reciprocal exchange between scaffold M and scaffold 33 and were found only in the CBS999.97(1-1, *re*) haploid genome. Accordingly, the *re* allele was likely to be evolved later in French Guiana. How such a chromosomal rearrangement occurred remains an open question. Genomic plasticity is often found in filamentous fungal species, and is thought to be a widely used strategy during fungal evolution [39]. In fact, different *Trichoderma* isolates also show variation in the karyotype [29,30]. Our data had identified a specific DNA rearrangement locus in the CBS999.97 wild isolate, and this information may provide a better picture in the study of the evolution of *Trichoderma* spp. Notably, the *re* locus may not be the only DNA rearrangement site within the *T. reesei* wild isolates. The sexual crossing experiment showed that the non-CBS999.97 wild isolates could not mate with each other, including those isolated from the same geographic areas [27] (Additional file 8: Table S4). However, they could all undergo sexual reproduction with the CBS999.97 strains (Table 2). Within these wild isolates, novel diversity may be found and linked to the evolution of sexual development genes.

Third, fungi have been reported to utilize different mechanisms to generate SAN progeny via meiosis, including hybrid infertility in *S. pombe* and *S. kambucha* [14] and telomere-telomere fusion and chromosomal translocation between two different chromosomes in *C. neoformans* [12,13]. Here, we showed that, due to a reciprocal DNA rearrangement between two scaffolds, the *T. reesei* CBS999.97 wild isolate produces both viable and inviable SAN progeny via meiosis. Our genetic results also revealed that interhomolog recombination or crossover occurred at a high frequency (>80%) to produce the “tetratype” asci (Figure 4B). Further investigation is needed to identify whether these crossover products are generated via a Spo11-induced or Spo11-independent DSB, where the DSB hotspot may be located, and if the DSB hotspot is created by a reciprocal exchange between scaffold M and scaffold 33.

Many *Trichoderma* species are found as anamorphs present in soils, where they act as plant beneficial fungi. In contrast, the teleomorphic *Hypocrea* species are most frequently found on decorticated wood or wood-rotting fungi (for example, wood ear fungi, shelf fungi, or agarics). With better lignocellulosic biomass degradation capability, the SAN sexual progeny we described in this report can likely provide adaptive advantages to the natural environments, especially in the early phase of colonization (the first two weeks of growth). Further study of the molecular mechanism leading to high hemicellulase production is thus of high interest. The D segment contains not only a few hemicellulase or xylanase encoding genes but also several novel transcription factors, including Gal4-like genes (ID: 70414, 111446, 111466, 36913) and fungal specific transcription factors (ID: 123860, 5664, 111515, and 69077) (Additional file 6: Table S2). Further investigation will reveal whether these transcription factors control the genome-wide expression of hemicellulase or xylanase encoding genes. Finally, for economical application, finding a way to prevent the high hemicellulase-producing SAN strains from RTU or at least to prolong the stability of these SAN strains is important. The very first step is to determine the molecular mechanism of RTU.

## Conclusion

*Trichoderma reesei* QM6a and its derivatives are industrial workhorse fungi that secrete cellulases and hemicellulases to degrade lignocellulosic biomass into glucose and xylose. CBS999.97 is the only *T. reesei* wild isolate strain that is sexually competent under laboratory conditions. Here we show that CBS999.97 sexual reproduction undergoes meiosis and two rounds of postmeiotic mitosis to yield asci with 16 linearly arranged ascospores. Notably, the two haploid genomes of the CBS999.97 wild isolate comprise a reciprocal arrangement between two chromosomal scaffolds. Due to sequence heterozygosity, most 16-spore asci contain four or eight inviable ascospores and an equal number of segmentally aneuploid (SAN) ascospores. The meiotic driven gene copy number change readily allows these viable SAN progeny to display new phenotypes, that is, white conidia, higher levels of hemicellulases, and genome instability. Our results have revealed a new understanding of *T. reesei* evolution and sexual development and also provided novel perspectives for improving industrial strains.

## Materials and methods

### Strains and sexual crossing

Sexual crossing was carried out as described previously [27,40]. The two parental haploids CBS999.97(1-1, *re*) and CBS999.97(1-2, *wt*) were generated from the CBS999.97 wild isolate strain [27,40]. The QM6a *tku70*Δ(1-2, *wt*) mutant [34] and the QM6a *tmus53*Δ(1-2, *wt*) mutant [33] were described previously. The *tku70*Δ and *tmus53*Δ mutants in the CBS999.97 background were generated by crossing each QM6a mutant with the wild isolate CBS999.97(1-1, *re*), respectively. The corresponding offspring mutants were backcrossed at least twice with the CBS999.97(1-2, *wt*) strains. All the strains used in this study are listed in Additional file 10: Table S5.

### Hexadecad dissection

Mature hexadecads were manually isolated from stromata and transferred onto the center of a 10-cm malt extract agar (MEA) plate. Yeast tetrad dissection using a micromanipulator was applied to sequentially separate and isolate each ascospore in a hexadecad (Figure 1A). The fiberglass needle could readily break the fragile ascus wall and separate each ascospore, leaving the remaining part intact.

### Deep sequencing and *de novo* assembly of the wild-type CBS999.97(1-2, *wt*) genome

The shotgun library for 454 Sequencing was prepared with 0.5 μg of genomic DNA from the wild isolate CBS999.97(1-2, *wt*) haploid using the GS Rapid Library Prep Kit following the manufacturer’s protocol (Roche 454; 454 Life Sciences, Branford, CT, USA). The resulting library was examined by the BioAnalyzer DNA Chip assay (Agilent Technologies; Santa Clara, CA, USA), and FAM fluorescence was quantified using a Modulus fluorometer (Turner Biosystems, Sunnyvale, CA, USA). Sequencing was performed on a GS FLX Titanium system in the High Throughput Sequencing Core Facility at the Biodiversity Research Center at Academia Sinica, Taiwan. Raw reads were obtained from 2.5 sequencing runs totaling 873 Mb, with median read lengths ranging from 351 to 454 nt among the five datasets. *De novo* assembly was performed using Newbler v.2.5.3 (Roche 454) on a single CPU. The draft genome assembly consisted of 1,087 contigs with sizes ranging from 500 bp to 404,555 bp, with an average contig and N50 size of 29,833 bp and 66,873 bp, respectively. Additional *de novo* assembly and gene annotation were conducted by an in-house computational core. The assembled CBS999.97(1-2, *wt*) genomic sequences are available online (http://140.109.32.39/~lab229/contig_info/index.php).

For evaluation of the deep sequencing results, the community annotation including the Gene Ontology classification is available from the *T. reesei* genome database v.2.0 (http://genome.jgi-psf.org/Trire2/Trire2.home.html). Annotation was performed using BLAST to search for orthologous genes of *Trichoderma reesei* v.2.0 and *Trichoderma virens* Gv29-8 v2.0. Gene sequences were downloaded from the Joint Genome Institute (JGI) database (http://genome.jgi-psf.org/) [25,41]; there are 9,143 genes in QM6a(1-2, *wt*) and 12,427 in *T. virens*. We identified 8,106 CBS999.97(1-2, *wt*) and QM6a(1-2, *wt*) orthologous genes with high sequence similarity (≥90%). Of the remaining genes (similarity <90%), we found 49 that existed only in QM6a(1-2, *wt*), without any similarity to genes in the CBS999.97(1-2, *wt*) genome.

### Array-based comparative genomic hybridization (aCGH) and data analysis

Genomic DNA was isolated using standard techniques and fragmented by a Bioruptor Sonicator (Diagenode, UCD-200) using repeated cycles of 75 seconds on (high) and then 75 seconds off for a total of 15 minutes, producing a median DNA size of 500 bp (range 200 to 1,000 bp). The fragmented DNA was then quantified using a NanoDrop ND-1000 UV-Vis Spectrophotometer to assess the genomic DNA concentration and purity. Fragmented genomic DNA samples were labeled with Cy5 or Cy3 using a NimbleGen Dual-Color DNA Labeling Kit (Roche NimbleGen, Madison, WI, USA). Test sample genomic DNA was end-labeled with Cy3, whereas CBS999.97(1-2, *wt*) genomic DNA was labeled with Cy5 and used as a reference to measure DNA copy number changes. The Cy5- and Cy3-labeled genomic DNA samples were hybridized to custom-designed oligonucleotide arrays (4 × 72,000 formation) by Roche-NimbleGen based on the CBS999.97(1-2, *wt*) genome sequence and *T. reesei* v2.0 genome sequence, respectively [40,42].

DNA end-labeling, hybridization, and scanning were performed by the Academia Sinica Institute of Molecular Biology Microarray Core using the NimbleGen Systems technique (NG_CGH&CNV_Guide_v7p0), following the vendor’s standard operating protocol. Image data were processed using NimbleScan software version 2.6.3 (Roche NimbleGen) to obtain the raw intensity data NimbleGen(.pair file). Data analysis and normalization were performed using Agilent GeneSpring GX 11.5.1 by an in-house bioinformatics core. Raw intensity scales were transformed by quantile normalization which was used to correct array biases and make all distributions uniform.

### Shake flask cultures

Conidia from three-day-old plates (10-cm diameter dishes) were harvested with 1 mL of sterile spore solution [0.8% NaCl, 0.05% Tween 20 (Sigma)], vortexed and filtered through glass wool. The volume was then adjusted to OD_600nm_ of 0.3, and 1 mL of the spore suspension was transferred to 250-mL Erlenmeyer flasks containing 50 mL of Mandels-Andreotti basal medium prepared in 0.1 M citrate-phosphate buffer (pH 5.0) and supplemented with 1 g L^-1^ peptone and 0.33 g L^-1^ urea. As a carbon source, 10 g L^-1^ Solka Floc 200 (International Fiber Corporation, North Tonawanda, NY, USA) or birchwood xylan (Sigma Aldrich, USA) was added to induce cellulase or xylanase expression, respectively. After three days of cultivation in constant darkness at 25°C on a rotary shaker (200 rpm), the flask culture was used for biomass determination and enzymatic assays.

### Biomass determination

For biomass determination, 50 mL shake flask culture was filtered onto a dry Miracloth (Calbiochem, Darmstadt, Germany), washed with distilled water, and dried with paper towels. The fungal mycelia were collected, frozen with liquid nitrogen, and stored at -80°C until use. For total protein quantification, the fungal sample was dissolved in 5 mL of 0.1 N NaOH. The solution was sonicated by a digital sonifier (Branson) at a duty cycle setting of 30% for six minutes total with 30 seconds on and 30 seconds off. The sample was then incubated at room temperature for three hours and centrifuged at 5251 × g for 10 minutes. The protein concentration of the supernatant was determined by modified Lowry protein assay, using bovine serum albumin as a standard.

### Enzymatic assays

For endo-1,4-β-glucanase (cellulase) activity measurements, supernatant diluted from 1:2 to 1:10 was added to an Azo-CM-cellulose solution (S-ACMCL; Megazyme International, Wicklow, Ireland). The procedure was carried out according to the manufacturer’s instructions. Azo-CM-cellulose is a dyed polysaccharide containing Remazol Brilliant Blue R at a concentration of approximately one dye molecule per 20 sugar residues (Megazyme International). This assay is much more sensitive (50- to 100-fold) than filter paper assays (such as the DNS method).

To measure endo-1,4-β-D-xylanase (xylanase) activity, supernatant diluted from 1:2 to 1:10 was prewarmed at 40°C for five minutes. A xylazyme AX test tablet substrate (T-XAX200; Megazyme International) was added to the supernatant (0.5 mL) and incubated at 40°C for 10 minutes. The reaction was stopped by adding 10 mL stop solution (2% Trizma Base, pH about 9). The sample was centrifuged at 2000 × g for 10 minutes, and the absorbance was measured at 590 nm. *Aspergillus niger* xylanase (about 300 mU/mL) was used as a control following the procedure specified by the manufacturer.

### Southern blot analysis

The Southern blot analysis procedure was followed using standard techniques. Genomic DNA was first digested by the indicated restriction enzyme, and 0.8% agarose gel was used for the gel electrophoresis. The DNA probes were amplified with indicated primers by PCR. The Southern blots were exposed to an X-ray film (GE Healthcare, USA).

### Rice straw growth assay

The conidia of the indicated strains were collected with Mandels-Andreotti basal medium (without carbohydrate supplement) and purified through glass wool. 2 × 10^6^ conidia were cultured on autoclaved 70 mm rice straw at 25°C, under conditions of 12 hours light/dark for five days. 1 mL of Mandels-Andreotti basal medium (without carbohydrate supplement) was added to the rice straw before the incubation.

### Microarray data access

The microarray data and the related protocols are available at the GEO site (http://www.ncbi.nlm.nih.gov/geo/) under accession numbers GSE-41965, GSE-42359, GSE-59350.

## Additional files

Additional file 1: Figure S1. Genotyping. Genomic PCR analysis of *mat1-1*, *mat1-2,* scaffold F, scaffold X, scaffold 33, scaffold M, and *tact* (actin) genes in all 16 viable ascospores of asci IV in Figure 1B. The parental wild isolate haploid strains, CBS999.97(1-1, *re*) and CBS999.97(1-2, *wt*), were used as controls. The nucleotide sequences of the PCR primers are listed in Additional file 2: Table S1. Additional file 2: Table S1. Oligonucleotide primers used in this study. Additional file 3: Figure S2. Visualization of four rounds of nuclear division during *T. reesei* ascospore formation/maturation. **(A-G)** Developing asci were manually dissected, stained with DAPI, and then visualized by fluorescent microscopy. DIC and DAPI fluorescent images are shown. Nuclei (N) are marked by white arrows. **(H)** A DIC image of developing asci showing synchronous division of eight nuclei (8 N) into 16 nuclei (16 N). Additional file 4: Figure S3. Scaffold M is a contiguous segment that includes the scaffolds 27, 28, and 36. **(A)** A representative aCGH result of viable SAN progeny. Normalized means of gene copy number for the oligonucleotides covering scaffold 25 to scaffold 37 are shown. These 13 scaffolds are ordered from left to right according to their length. **(B)** A schema illustrates the order of scaffold 28(3′), scaffold 27, scaffold 36, and scaffold 28(5′) in the scaffold M. The locations of six PCR primers (E, F, G, H, I, and J; see Additional file 2: Table S1) in the scaffold M are indicated. **(C)** Genomic PCR. The genomic DNA of two parental haploids strains, CBS999.97(1-1, *re*) (1) and CBS999.97(1-2, *wt*) (2), were amplified using three pairs of PCR primers (E/F, G/H, and I/J). The expected PCR products are 7,168 bp, 1,000 bp, and 1,272 bp in length, respectively. The *tact* (actin) gene was used as positive control for genomic PCR. Additional file 5: Figure S4. Differential chromosomal organization in the genomes of QM6a(1-2, *wt*), CBS999.97(1-1, *re*), and CBS999.97(1-2, *wt*), respectively. **(A)** Scaffold 33 and scaffold M in QM6a(1-2, *wt*) and CBS999.97(1-2, *wt*). Scaffold 27, scaffold 28, scaffold 33, and scaffold 36 are indicated. The 5′ terminus of scaffold M has a telomere (in black), because the corresponding end in scaffold 28(3′) is connected to multiple copies of a repeated hexanucleotide sequence, TTAGGG, which is the telomeric repeat of QM6a(1-2, *wt*) [25]. The D segment and the S segment of scaffold M are indicated in orange and green, respectively. The L segment, located at the 5′ terminus of scaffold 33, is indicated in light gray, and the remaining portion of scaffold 33, the N segment, is indicated in dark gray. The three exons (E1, E2, and E3) of a novel gene (ID: 112288) in scaffold 36 are indicated. The first exon (E1) only exists in the QM6a(1-2, *wt*) genome. **(B)** Scaffold F and scaffold X in CBS999.97(1-1, *re*). The nucleotide sequences of scaffold M, scaffold 33, scaffold F, and scaffold X are available online (http://bc.imb.sinica.edu.tw/~lab229/Text_file_T1-4.rar). **(C)** Schema illustrating the location of PCR primers (A, B, C, and D) used for the genotyping scaffold M, scaffold F, scaffold 33, and scaffold X, respectively. The nucleotide sequences of these four primers are listed in Additional file 2: Table S1. **(D)** PCR genotyping of indicated haploid strains. The two parental haploid strains, CBS999.97(1-1, *re*) and CBS999.97(1-2, *wt*), were used as positive controls. CBS999.97(1-1, *wt*) and CBS999.97(1-2, *re*) are progeny generated by sexually crossing CBS999.97(1-1, *re*) with CBS999.97(1-2, *wt*). The corresponding progeny in *tku70*Δ and *tmus53*Δ were also generated by sexually crossing, respectively (Additional file 10: Table S5). Additional file 6: Table S2. Genes on the D segment. Additional file 7: Table S3. Genomic PCR genotyping the four scaffolds (M, F, 33, X) in *T. reesei* wild isolates and industrial strains. Additional file 8: Table S4. Sexual crossing of the non-CBS999.97 isolates. Additional file 9: Figure S5. Hexadecad dissection of asci generated from sexual crossing the CBS999.97(1-1, *re*) with RUT-C30(1-2, *wt*) or QM9414(1-2, *wt*). Hexadecads from sexual crossing of CBS999.97(1-1, *re*) with RUT-C30(1-2, *wt*) (n ≥ 10) **(A)** or QM9414(1-2, *wt*) (n ≥ 10) **(B)**. Sixteen ascospores from each hexadecad were sequentially separated and aligned as described in Figure 1A. The inviable ascospores are indicated by a black circle with a cross. Additional file 10: Table S5. Strains used in this study.

## Abbreviations

aCGH: array-based comparative genomic hybridization
DAPI: 4',6-diamidino-2-phenylindole
DIC: differential interference contrast
DSB: double-strand break
RTU: return to euploidy
SAN: segmentally aneuploid
