# Supplementary figures and images for: Trichoderma reesei meiosis generates segmentally aneuploid progeny with higher xylanase-producing capability
Source: Biotechnol Biofuels. 2015 Feb 25;8:30. doi: 10.1186/s13068-015-0202-6 (PMC4344761; doi:10.1186/s13068-015-0202-6)

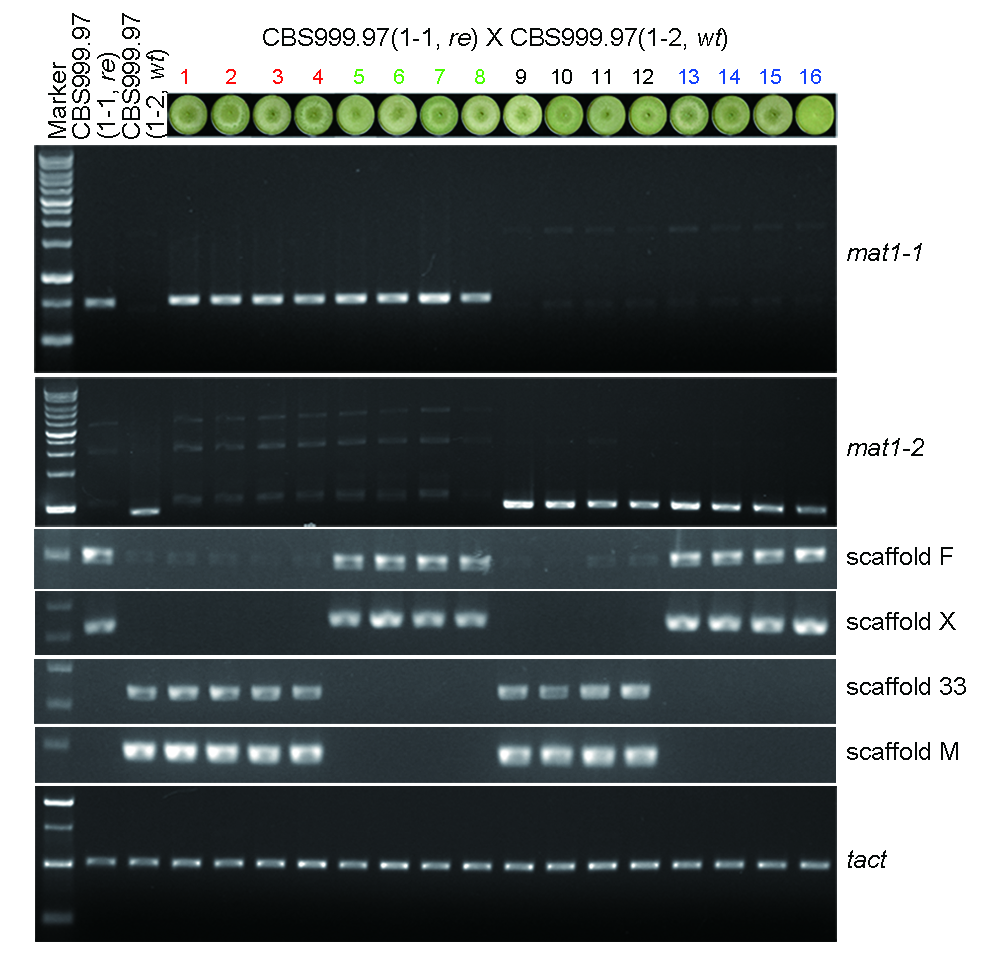

Supplement: Additional file 1: Figure S1. — Genotyping. Genomic PCR analysis of mat1-1, mat1-2, scaffold F, scaffold X, scaffold 33, scaffold M, and tact (actin) genes in all 16 viable ascospores of asci IV in Figure 1B. The parental wild isolate haploid strains, CBS999.97(1-1, re) and CBS999.97(1-2, wt), were used as controls. The nucleotide sequences of the PCR primers are listed in Additional file 2: Table S1. [file 13068_2015_202_MOESM1_ESM.tif]

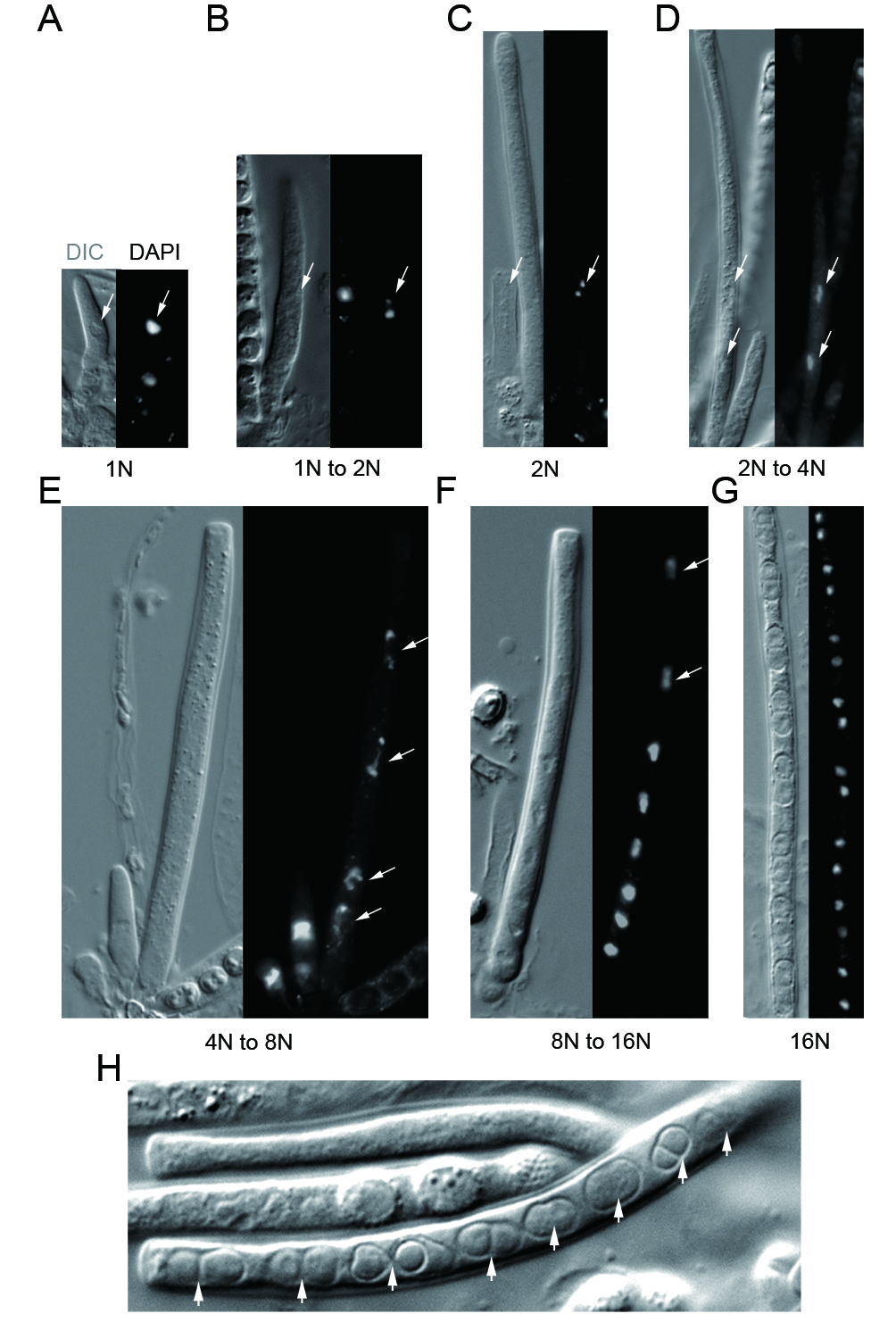

Supplement: Additional file 3: Figure S2. — Visualization of four rounds of nuclear division during T. reesei ascospore formation/maturation. (A-G) Developing asci were manually dissected, stained with DAPI, and then visualized by fluorescent microscopy. DIC and DAPI fluorescent images are shown. Nuclei (N) are marked by white arrows. (H) A DIC image of developing asci showing synchronous division of eight nuclei (8 N) into 16 nuclei (16 N). [file 13068_2015_202_MOESM3_ESM.tif]

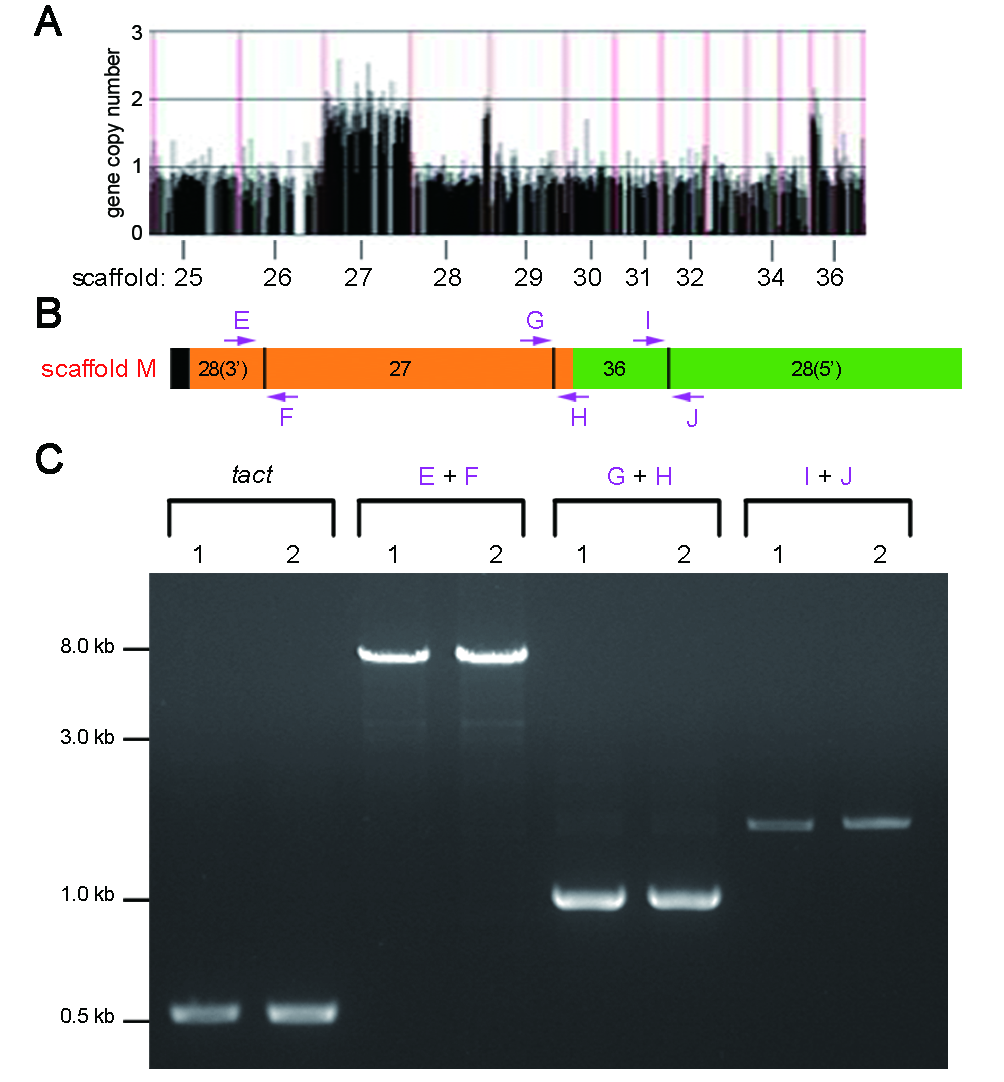

Supplement: Additional file 4: Figure S3. — Scaffold M is a contiguous segment that includes the scaffolds 27, 28, and 36. (A) A representative aCGH result of viable SAN progeny. Normalized means of gene copy number for the oligonucleotides covering scaffold 25 to scaffold 37 are shown. These 13 scaffolds are ordered from left to right according to their length. (B) A schema illustrates the order of scaffold 28(3′), scaffold 27, scaffold 36, and scaffold 28(5′) in the scaffold M. The locations of six PCR primers (E, F, G, H, I, and J; see Additional file 2: Table S1) in the scaffold M are indicated. (C) Genomic PCR. The genomic DNA of two parental haploids strains, CBS999.97(1-1, re) (1) and CBS999.97(1-2, wt) (2), were amplified using three pairs of PCR primers (E/F, G/H, and I/J). The expected PCR products are 7,168 bp, 1,000 bp, and 1,272 bp in length, respectively. The tact (actin) gene was used as positive control for genomic PCR. [file 13068_2015_202_MOESM4_ESM.tif]

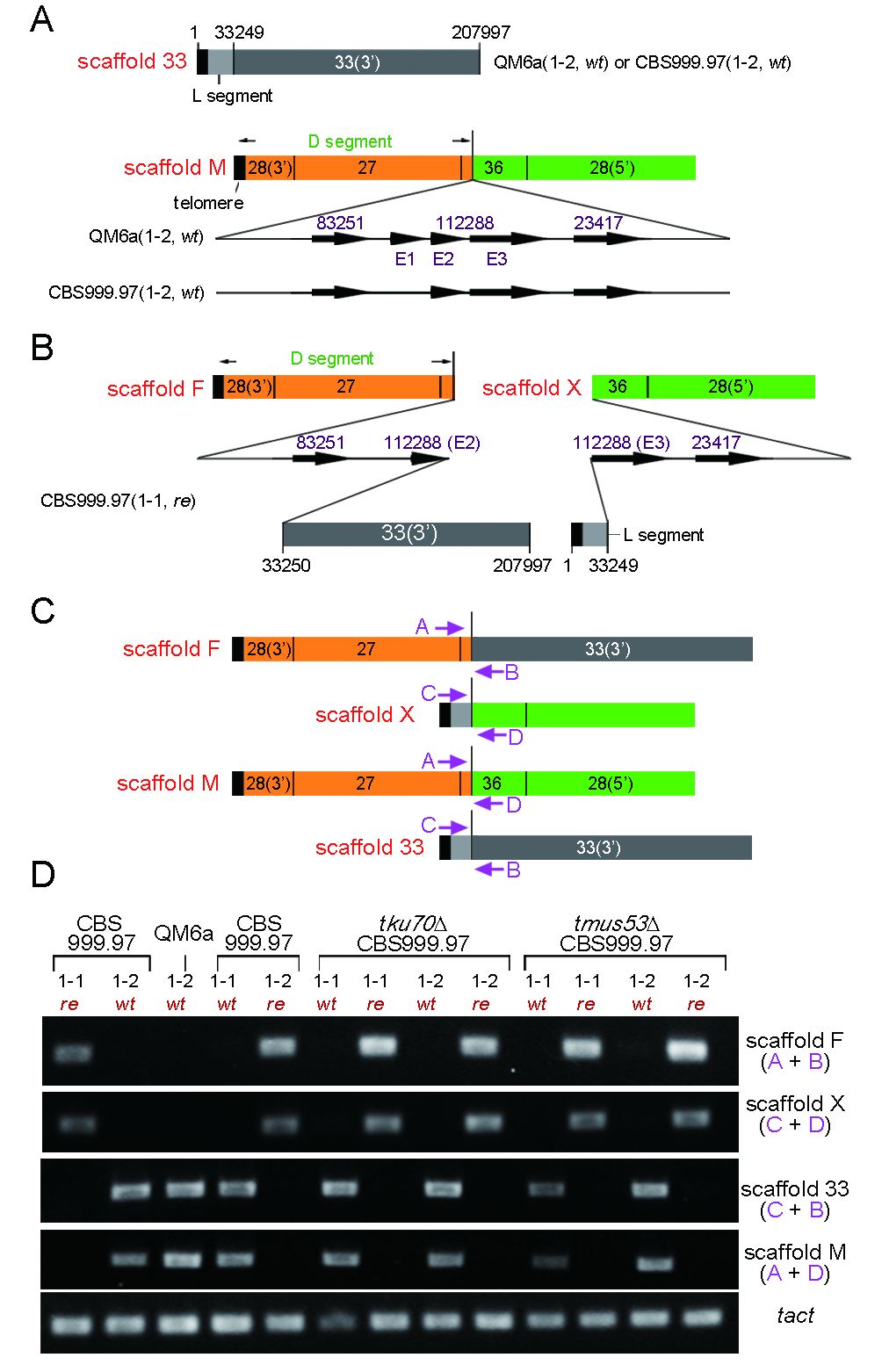

Supplement: Additional file 5: Figure S4. — Differential chromosomal organization in the genomes of QM6a(1-2, wt), CBS999.97(1-1, re), and CBS999.97(1-2, wt), respectively. (A) Scaffold 33 and scaffold M in QM6a(1-2, wt) and CBS999.97(1-2, wt). Scaffold 27, scaffold 28, scaffold 33, and scaffold 36 are indicated. The 5′ terminus of scaffold M has a telomere (in black), because the corresponding end in scaffold 28(3′) is connected to multiple copies of a repeated hexanucleotide sequence, TTAGGG, which is the telomeric repeat of QM6a(1-2, wt) [25]. The D segment and the S segment of scaffold M are indicated in orange and green, respectively. The L segment, located at the 5′ terminus of scaffold 33, is indicated in light gray, and the remaining portion of scaffold 33, the N segment, is indicated in dark gray. The three exons (E1, E2, and E3) of a novel gene (ID: 112288) in scaffold 36 are indicated. The first exon (E1) only exists in the QM6a(1-2, wt) genome. (B) Scaffold F and scaffold X in CBS999.97(1-1, re). The nucleotide sequences of scaffold M, scaffold 33, scaffold F, and scaffold X are available online (http://bc.imb.sinica.edu.tw/~lab229/Text_file_T1-4.rar). (C) Schema illustrating the location of PCR primers (A, B, C, and D) used for the genotyping scaffold M, scaffold F, scaffold 33, and scaffold X, respectively. The nucleotide sequences of these four primers are listed in Additional file 2: Table S1. (D) PCR genotyping of indicated haploid strains. The two parental haploid strains, CBS999.97(1-1, re) and CBS999.97(1-2, wt), were used as positive controls. CBS999.97(1-1, wt) and CBS999.97(1-2, re) are progeny generated by sexually crossing CBS999.97(1-1, re) with CBS999.97(1-2, wt). The corresponding progeny in tku70Δ and tmus53Δ were also generated by sexually crossing, respectively (Additional file 10: Table S5). [file 13068_2015_202_MOESM5_ESM.tif]

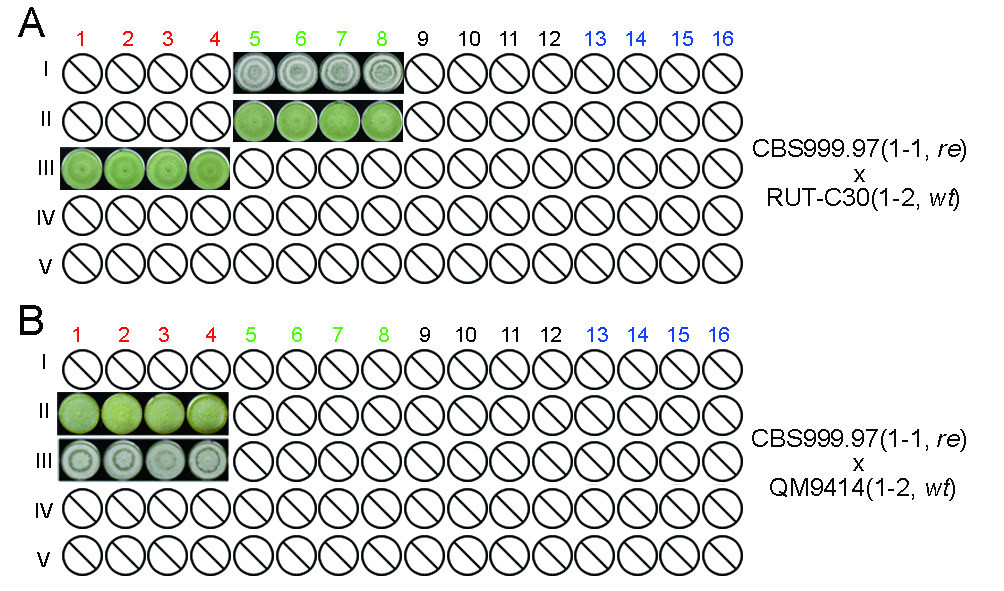

Supplement: Additional file 9: Figure S5. — Hexadecad dissection of asci generated from sexual crossing the CBS999.97(1-1, re) with RUT-C30(1-2, wt) or QM9414(1-2, wt). Hexadecads from sexual crossing of CBS999.97(1-1, re) with RUT-C30(1-2, wt) (n ≥ 10) (A) or QM9414(1-2, wt) (n ≥ 10) (B). Sixteen ascospores from each hexadecad were sequentially separated and aligned as described in Figure 1A. The inviable ascospores are indicated by a black circle with a cross. [file 13068_2015_202_MOESM9_ESM.tif]
