# Supplementary material for: Trichoderma reesei meiosis generates segmentally aneuploid progeny with higher xylanase-producing capability
Source: Biotechnol Biofuels. 2015 Feb 25;8:30. doi: 10.1186/s13068-015-0202-6 (PMC4344761; doi:10.1186/s13068-015-0202-6)
Supplement: Additional file 2: Table S1. — Oligonucleotide primers used in this study. [file 13068_2015_202_MOESM2_ESM.pdf]

Additional file 2: Table S1
Oligonucleotide primers used in this study.

Protein ID or location	Primer name	Nucleotide sequences	Remarks
44504 (*tact*)	PA6693	5'ATGATCGGTATGGGTCAG3'	Figure 6D, S1, S3C & S4D
	PA6694	5'GATGTCACGGACGATTTC3'	
Genebank accession number FJ599756 (*mat1-1*)	PA6066	5'AGCCGAGATACCTCAATG3'	Figure 6D, S1
	PA6067	5'ACCTGTCCTCCAATCTTC3'	
124341 (*mat1-2*)	PA6068	5'TCAGTCAACGCAGTCATG3'	Figure 6D, S1
	PA6069	5'CATTGGCACAAGCGAC3'	
82208 (*tpks4*)	PA7809	5'TCATGCGTCGCACATCTTCT3'	Figure 6D
	PA7810	5'TCCAGCAACCATGCATGATC3'	
3′ D segment	A	5'CTTCCAGCCTAAGTACTC3'	Figure S1 & S4
5′ N segment	B	5'GTCGATCGTGCTAATGAAG3'	Figure S1 & S4
3′ L segment	C	5'CAAGGCTATTATCCGCAG3'	Figure S1 & S4
5′ S segment	D	5'CTCTGAGGGGATTAGAAG3'	Figure S1 & S4
3′ scaffold 28 (3′)	E	5'CCAAGCCTGATATGCTGT3'	Figure S3
5′ scaffold 27	F	5'GAGTATCAGTCCGCCAGA3'	Figure S3
3′ scaffold 27	G	5'TCCAGGAACAGCCATAGA3'	Figure S3
5′ scaffold 36	H	5'GCAGATGCCTTGTTCTGT3'	Figure S3
3′ scaffold 36	I	5'TACTTCCCTGCAGGTTTA3'	Figure S3
5′ scaffold 28(5’)	J	5'AGCCCCTGCCTTATTTTA3'	Figure S3
3′ D segment (DNA probe)	PA7543	5'AGCTACATGAGATCCTGCAT3'	Figure 6B
	PA7544	5'TTCGTGCAGAGTGCACCTGA3'	
