# Supplementary material for: Trichoderma reesei meiosis generates segmentally aneuploid progeny with higher xylanase-producing capability
Source: Biotechnol Biofuels. 2015 Feb 25;8:30. doi: 10.1186/s13068-015-0202-6 (PMC4344761; doi:10.1186/s13068-015-0202-6)
Supplement: Additional file 6: Table S2. — Genes on the D segment. [file 13068_2015_202_MOESM6_ESM.pdf]

Additional file 6: Table S2
Genes on the D Segment. *

Protein ID	Note
81955	Electron transport
69210	Electron transport
69291	Electron transport
111724	Major facilitator superfamily
69245	Beta-mannosidase, GH2
123940	Cellulose-binding region, fungal
69276	Beta-glucosylceramidase, GH30
111716	Cinnamyl-alcohol dehydrogenase activity
36913	Fungal specific transcription factor
68950	Electron transport
68961	Esterase/lipase/thioesterase
69168	Isoflavone reductase
123801	tRNA synthetase, class II
81700	Mannose-6-phosphate isomerase, type I
111440	Nucleolar GTPase/ATPase p130
123805	GTP cyclohydrolase II
123806	G-protein coupled receptor
111446	Fungal transcriptional regulatory protein
111447	Sulfatase
27939	Major facilitator superfamily, Sugar transporter
111449	Cerato-platanin
111451	Peptidase
51967	Translational termination
69164	Monocarboxylate transporter
68941	Glutathione S-transferase
123809	Major facilitator superfamily, Sugar transporter
81713	Ribosomal protein
5633	Predicted methyltransferase
81716	RNA-binding region RNP-1 (RNA recognition motif)
111466	Fungal transcriptional regulatory protein
41942	Asp/Glu racemase
111468	Actin-repated protein Arp2
81720	Cyclin C-dependent kinase CDK8
23263	GTP-binding ADP-ribosylation factor Arf6 (dArf3)
69118	Histone acetyltransferase SAGA associated factor SGF29
111476	Peptidase
123818	Xylasase, GH11
69061	Predicted phosphoglycerate mutase
69035	Cytidylyltransferase
123820	Aminotransferase, class IV
68966	Flavoprotein

81730	Electron transport
5645	Gamma-glutamyltranspeptidase
68956	D-aspartate oxidase
68924	SAM-dependent methyltransferases
69066	catalytic activity
123827	Haem peroxidase
69171	Zinc-containing alcohol dehydrogenase
111499	Oxidoreductase activity
69115	Dienelactone hydrolase
111502	NADH-ubiquinone oxidoreductase B18 subunit
23268	Forkhead-associated
81742	DNA-dependent RNA polymerase I
68972	Major facilitator superfamily, sugar transporter
68925	Major facilitator superfamily, Synaptic vesicle transporter SVOP
69055	Isochorismatase hydrolase
69077	Fungal transcriptional regulatory protein
5656	Major facilitator superfamily, sugar transporter
123831	Serine/threonine protein kinase
68973	Metallophosphoesterase
111515	Fungal specific transcription factor
123832	Molecular chaperone (DnaJ superfamily)
5659	mRNA splicing factor ATP-dependent RNA helicase
52012	Prolyl 4-hydroxylase, alpha subunit
111523	C-type lectin
23271	COPII vesicle protein
69123	Mannan endo-1,6-alpha-mannosidase activity, GH76
123837	Actin and related proteins
5664	Transcription factor
81757	Histone acetyltransferase activity
52021	MEKK and related serine/threonine protein kinases
111536	Folylpolyglutamate synthetase
69064	Uncharacterized conserved protein
111538	Hemopexin
68988	Major facilitator superfamily
111541	Glucose/ribitol dehydrogenase
69141	amino acid transport
69122	Fatty acid desaturase
23276	VAMP-associated protein involved in inositol metabolism
69068	Predicted ubiquitin regulatory protein
123850	Ribosomal protein 60S
68926	Cdc42-interacting protein CIP4
5675	Zn-finger, C2H2 type
68990	Major facilitator superfamily
81783	Serine proteinase inhibitor
111561	Transcription factors

81785	GTPase-activating protein
52050	HSP90 co-chaperone CPR7/Cyclophilin
111564	D-Tyr-tRNA (Tyr) deacylase
123860	Transcription factors
111567	Zn-finger, C2H2 type
111569	Myosin class II heavy chain
111570	Glucose/ribitol dehydrogenase
52055	NADP-dependent isocitrate dehydrogenase
23287	U1 snRNP complex
81797	Vesicle-mediated transport
111575	Nuclear receptor coregulator SMRT/SMRTER
69052	Translin family protein
123865	Peptidase
81803	Predicted member of the intramitochondrial sorting protein family
81804	Metallopeptidase
69153	Pantothenate kinase activity
69013	Uncharacterized conserved protein
69081	Glycolate oxidase
68997	TPR repeat
23292	Zinc-binding oxidoreductase
112271	Chitin biosynthesis, Glycosyl transferase, family 2
70429	Predicted dehydrogenase
70414	Fungal transcriptional regulatory protein
23415	Amino acid transporters
70491	Esterase/lipase/thioesterase
112283	Dynactin, subunit p25
82351	Mitogen-activated protein kinase

* Annotations was performed using SEARCH to search for the protein ID of the *Trichoderma reesei* v.2.0 (http://genome.jgi-psf.org/Trire2/Trire2.home.html) of the Joint Genome Institute (JGI) database (http://genome.jgi-psf.org/) [25, 41].
