# Supplementary material for: Trichoderma reesei meiosis generates segmentally aneuploid progeny with higher xylanase-producing capability
Source: Biotechnol Biofuels. 2015 Feb 25;8:30. doi: 10.1186/s13068-015-0202-6 (PMC4344761; doi:10.1186/s13068-015-0202-6)
Supplement: Additional file 7: Table S3. — Genomic PCR genotyping the four scaffolds (M, F, 33, X) in T. reesei wild isolates and industrial strains. [file 13068_2015_202_MOESM7_ESM.pdf]

Additional file 7: Table S3
Genomic PCR genotyping the four scaffolds (M, F, 33, X) in *T. reesei* wild isolates and industrial strains.

Wild isolate	Origin	Mating type	PCR primers A + B (F)	PCR primers C+ D (X)	PCR primers C + B (33)	PCR primers A + D (M)	*re*
CBS999.97	French Guiana	*MAT1-1*	+	+	-	-	+
CBS999.97	French Guiana	*MAT1-2*	-	-	+	+	-
QM6a	Solomon Islands	*MAT1-2*	-	-	+	+	-
G.J.S. 86-404	French Guiana	*MAT1-1*	-	-	+	+	-
G.J.S. 86-410	French Guiana	*MAT1-1*	-	-	+	-	-
G.J.S. 84-473	French Guiana	*MAT1-1*	-	-	-	-	-
G.J.S. 89-7	Brazil Para	*MAT1-2*	-	-	+	+	-
G.J.S .97-178	Brazil Para	*MAT1-2*	-	-	+	+	-
G.J.S. 85-249	Indonesia Celebes	*MAT1-1*	-	-	+	+	-
G.J.S. 85-229	Indonesia Celebes	*MAT1-2*	-	-	+	+	-
G.J.S. 85-236	Indonesia Celebes	*MAT1-2*	-	-	+	+	-
G.J.S. 93-23	New Caledonia	*MAT1-2*	-	-	+	-	-
QM9414		*MAT1-2*	-	-	+	+	-
RUT-C30		*MAT1-2*	-	-	+	+	-
