# Supplementary material for: Trichoderma reesei meiosis generates segmentally aneuploid progeny with higher xylanase-producing capability
Source: Biotechnol Biofuels. 2015 Feb 25;8:30. doi: 10.1186/s13068-015-0202-6 (PMC4344761; doi:10.1186/s13068-015-0202-6)
Supplement: Additional file 8: Table S4. — Sexual crossing of the non-CBS999.97 isolates. [file 13068_2015_202_MOESM8_ESM.pdf]

Additional file 8: Table S4
Sexual crossing of the non-CBS999.97 isolates.

Sexual crossing	Fruiting body
G.J.S. 86-410 (1-1, *wt*; French Guiana) & G.J.S. 89-7 (1-2, *wt*; Brazil, Para)	-
G.J.S. 86-410 (1-1, *wt*; French Guiana) & G.J.S. 85-229 (1-2, *wt*; Indonesia, Celebes)	-
G.J.S. 84-473 (1-1, *wt*; French Guiana) & G.J.S. 85-229 (1-2, *wt*; Indonesia, Celebes)	-
G.J.S. 84-473 (1-1, *wt*; French Guiana) & G.J.S. 93-23 (1-2, *wt*; New Caledonia)	-
G.J.S. 85-249 (1-1, *wt*; Indonesia, Celebes) & G.J.S. 85-229 (1-2, *wt* ;Indonesia, Celebes)	-
G.J.S. 85-249 (1-1, *wt*; Indonesia, Celebes) & G.J.S. 85-236 (1-2, *wt*; Indonesia, Celebes)	-
G.J.S. 85-249 (1-1, *wt*; Indonesia, Celebes) & G.J.S. 93-23 (1-2, *wt*; New Caledonia)	-
