# Supplementary material for: Trichoderma reesei meiosis generates segmentally aneuploid progeny with higher xylanase-producing capability
Source: Biotechnol Biofuels. 2015 Feb 25;8:30. doi: 10.1186/s13068-015-0202-6 (PMC4344761; doi:10.1186/s13068-015-0202-6)
Supplement: Additional file 10: Table S5. — Strains used in this study. [file 13068_2015_202_MOESM10_ESM.pdf]

Additional file 10: Table S5
Strains used in this study.

Strain	Description	References & remarks
WTH0005	QM9414(1-2, *wt*)	[22, 23]
WTH0007	QM6a(1-2, *wt*)	[36]
WTH0011	CBS999.97(1-1, *re*)	[27, 35]
WTH0013	CBS999.97(1-2, *wt*); E0-Euploidy (Figure 2)	[27, 35]
WTH0249	QM6a *tku70Δ* (1-2, *wt*)	[34]
WTH0270	CBS999.97(1-1, *wt*)<br>An ascospore from sexual crossing WTH0011 with WTH0013 (ascospore #1 of acsi III in Figure 1B)	Figure 2B, S1 & S4D<br>Table 1 & 2
WTH0271	CBS999.97(1-1, *wt*)<br>An ascospore from sexual crossing WTH0011 with WTH0013 (ascospore #2 of acsi III in Figure 1B)	Figure 2B & S1
WTH0272	CBS999.97(1-1, *wt*)<br>An ascospore from sexual crossing WTH0011 with WTH0013 (ascospore #3 of acsi III in Figure 1B)	Figure 2B & S1
WTH0273	CBS999.97(1-1, *wt*)<br>An ascospore from sexual crossing WTH0011 with WTH0013 (ascospore #4 of acsi III in Figure 1B)	Figure 2B & S1
WTH0274	CBS999.97(1-1, *re*)<br>An ascospore from sexual crossing WTH0011 with WTH0013 (ascospore #5 of acsi III in Figure 1B)	Figure 2B & S1
WTH0275	CBS999.97(1-1, *wt*)<br>An ascospore from sexual crossing WTH0011 with WTH0013 (ascospore #6 of acsi III in Figure 1B)	Figure 2B & S1
WTH0276	CBS999.97(1-1, *re*)<br>An ascospore from sexual crossing WTH0011 with WTH0013 (ascospore #7 of acsi III in Figure 1B)	Figure 2B & S1
WTH0277	CBS999.97(1-1, *re*)<br>An ascospore from sexual crossing WTH0011 with WTH0013 (ascospore #8 of acsi III in Figure 1B)	Figure 2B & S1
WTH0278	CBS999.97(1-2, *wt*)<br>An ascospore from sexual crossing WTH0011 with WTH0013 (ascospore #9 of acsi III in Figure 1B)	Figure 2B & S1
WTH0279	CBS999.97(1-2, *wt*)<br>An ascospore from sexual crossing WTH0011 with WTH0013 (ascospore #10 of acsi III in Figure 1B)	Figure 2B & S1
WTH0280	CBS999.97(1-2, *wt*)<br>An ascospore from sexual crossing WTH0011 with WTH0013 (ascospore #11 of acsi III in Figure 1B)	Figure 2B & S1
WTH0281	CBS999.97(1-2, *wt*)<br>An ascospore from sexual crossing WTH0011 with WTH0013 (ascospore #12 of acsi III in Figure 1B)	Figure 2B & S1

WTH0282	CBS999.97(1-2, *re*)<br>An ascospore from sexual crossing WTH0011 with WTH0013 (ascospore #13 of acsi III in Figure 1B)	Figure 2B, S1 & S4D<br>Table 1 & 2
WTH0283	CBS999.97(1-2, *re*)<br>An ascospore from sexual crossing WTH0011 with WTH0013 (ascospore #14 of acsi III in Figure 1B)	Figure 2B & S1
WTH0284	CBS999.97(1-2, *re*)<br>An ascospore from sexual crossing WTH0011 with WTH0013 (ascospore #15 of acsi III in Figure 1B)	Figure 2B & S1
WTH0285	CBS999.97(1-2, *re*)<br>An ascospore from sexual crossing WTH0011 with WTH0013 (ascospore #16 of acsi III in Figure 1B)	Figure 2B & S1
WTH0250	D1-SAN<br>An ascospore from sexual crossing WTH0011 with WTH0013 (ascospore #1 of asci IV in Figure 1B)	Figure 2C
WTH0254	D2-SAN<br>An ascospore from sexual crossing WTH0011 with WTH0013 (ascospore #8 of asci IV in Figure 1B)	Figure 2C, 5, 6A, 6B & 7
WTH0258	N1-Euploidy<br>An ascospore from sexual crossing WTH0011 with WTH0013 (ascospore #1 of asci I in Figure 1B)	Figure 2D
WTH0262	D3-SAN<br>An ascospore from sexual crossing WTH0011 with WTH0013 (ascospore #9 of asci I in Figure 1B)	Figure 2D, 5 & 7
WTH0266	N2-Euploidy<br>An ascospore from sexual crossing WTH0011 with WTH0013 (ascospore #13 of asci I in Figure 1B)	Figure 2D, 7A & 7B
WTH0377	D5-SAN<br>An ascospore from sexual crossing WTH0011 with WTH0013	Figure 5, 6A, 6B & 7
WTH0608	RUT-C30(1-2, *wt*)	[21]
WTH0788	QM6a *tmus53*Δ (1-2, *wt*)<br>A gift from Astrid R. Mach-Aigner	[33]
WTH1861	CBS999.97 *tmus53*Δ (1-2, *wt*)	Figure S4D<br>Table 1
WTH1869	CBS999.97 *tmus53*Δ (1-1, *re*)	Figure S4D<br>Table 1
WTH1889	CBS999.97 *tmus53*Δ (1-2, *re*)	Figure S4D<br>Table 1
WTH1942	CBS999.97 *tmus53*Δ (1-1, *wt*)	Figure S4D<br>Table 1
WTH2036	CBS999.97 *tku70*Δ (1-1, *re*)	Figure S4D<br>Table 1
WTH3815	CBS999.97 *tku70*Δ (1-2, *re*)	Figure S4D

		Table 1
WTH3819	CBS999.97 *tku70*Δ (1-1, *wt*)	Figure S4D<br>Table 1
WTH3836	CBS999.97 *tku70*Δ (1-2, *wt*)	Figure S4D<br>Table 1
WTH4313	CBS999.97 D2-RTU<br>WTH0254 cultured in MEA for over 34 days	Figure 5, 6A, 6B, 6D & 7C
WTH4317	CBS999.97 D5-RTU<br>WTH0377 cultured in MEA for over 34 days	Figure 5, 6A, 6B & 7C
WTH4865	CBS999.97(1-2)<br>An ascospore from sexual crossing WTH4313 with WTH0013 (ascospore #1 in Fig 6D.)	Figure 6D & E
WTH4866	CBS999.97(1-2)<br>An ascospore from sexual crossing WTH4313 with WTH0013 (ascospore #2 in Figure 6D)	Figure 6D
WTH4867	CBS999.97(1-2)<br>An ascospore from sexual crossing WTH4313 with WTH0013 (ascospore #3 in Figure 6D)	Figure 6D
WTH4868	CBS999.97(1-2)<br>An ascospore from sexual crossing WTH4313 with WTH0013 (ascospore #4 in Figure 6D)	Figure 6D
WTH4869	CBS999.97(1-2)<br>An ascospore from sexual crossing WTH4313 with WTH0013 (ascospore #5 in Figure 6D)	Figure 6D & E
WTH4870	CBS999.97(1-2)<br>An ascospore from sexual crossing WTH4313 with WTH0013 (ascospore #6 in Figure 6D)	Figure 6D
WTH4871	CBS999.97(1-2)<br>An ascospore from sexual crossing WTH4313 with WTH0013 (ascospore #7 in Figure 6D)	Figure 6D
WTH4872	CBS999.97(1-2)<br>An ascospore from sexual crossing WTH4313 with WTH0013 (ascospore #8 in Figure 6D)	Figure 6D
WTH4873	CBS999.97(1-1)<br>An ascospore from sexual crossing WTH4313 with WTH0013 (ascospore #9 in Figure 6D)	Figure 6D & E
WTH4874	CBS999.97(1-1)<br>An ascospore from sexual crossing WTH4313 with WTH0013 (ascospore #10 in Figure 6D)	Figure 6D
WTH4875	CBS999.97(1-1)<br>An ascospore from sexual crossing WTH4313 with WTH0013 (ascospore #11 in Figure 6D)	Figure 6D
WTH4876	CBS999.97(1-1)<br>An ascospore from sexual crossing WTH4313 with WTH0013 (ascospore #12 in Figure 6D)	Figure 6D
WTH4877	CBS999.97(1-1)	Figure 6D & E

	An ascospore from sexual crossing WTH4313 with WTH0013 (ascospore #13 in Figure 6D)	
WTH4878	CBS999.97(1-1)<br>An ascospore from sexual crossing WTH4313 with WTH0013 (ascospore #14 in Figure 6D)	Figure 6D
WTH4879	CBS999.97(1-1)<br>An ascospore from sexual crossing WTH4313 with WTH0013 (ascospore #15 in Figure 6D)	Figure 6D
WTH4880	CBS999.97(1-1)<br>An ascospore from sexual crossing WTH4313 with WTH0013 (ascospore #16 in Figure 6D)	Figure 6D
WTH5089	G.J.S. 86-404 (1-1, *wt*; French Guiana)	[27]<br>Table 2, S3 & S4
WTH5090	G.J.S. 86-410 (1-1, *wt*; French Guiana)	[27]<br>Table 2, S3 & S4
WTH5091	G.J.S. 84-473 (1-1, *wt*; French Guiana))	[27]<br>Table 2, S3 & S4
WTH5092	G.J.S. 89-7 (1-2, *wt*; Brazil, Para)	[27]<br>Table 2, S3 & S4
WTH5093	G.J.S. 97-178 (1-2, *wt*; Brazil, Para)	[27]<br>Table 2, S3 & S4
WTH5094	G.J.S. 85-249 (1-1, *wt*; Indonesia, Celebes)	[27]<br>Table 2, S3 & S4
WTH5095	G.J.S. 85-229 (1-2, *wt*; Indonesia, Celebes)	[27]<br>Table 2, S3 & S4
WTH5096	G.J.S. 85-236 (1-2, *wt*; Indonesia, Celebes)	[27]<br>Table 2, S3 & S4
WTH5097	G.J.S. 93-23 (1-2, *wt*; New Caledonia)	[27]<br>Table 2, S3 & S4
